# Anthelmintic Potential of Conjugated Long-Chain Fatty Acids Isolated from the Bioluminescent Mushroom *Neonothopanus gardneri*

**DOI:** 10.1021/acs.jnatprod.4c00546

**Published:** 2025-01-04

**Authors:** Maria D. A. Oliveira, Teresinha de Jesus A. dos S. Andrade, Joaquim S. C. Junior, Nerilson Marques Lima, Hugo G. Machado, Jioji N. Tabudravu, Francisco das Chagas L. Pinto, Lucas Fukui-Silva, Monique C. Amaro, Josué de Moraes, Dulce Helena S. Silva, Antônia Maria das Graças L. Citó, Chistiane Mendes Feitosa

**Affiliations:** † Department of Chemistry, Federal University of Piaui, Campus Ministro Petrônio Portela, Teresina, PI 64049-550, Brazil; ‡ Nucleus of Applied Research to Sciences-NIAC, Federal Institute of Education, Science and Technology of Maranhao-IFMA, Presidente Dutra (Maranhao), Timon, Maranhao 65635-468, Brazil; § Department of Chemistry, Federal Institute of Piaui, Campus Central, Praça da Liberdade, Teresina, PI 64049-550, Brazil; ∥ School of Natural Sciences, Faculty of Science and Technology, University of Central Lancashire, PR1 2HE Preston, U.K.; ϕ Institute of Chemistry, Federal University of Alfenas, Alfenas MG 37130-001, Brazil; ⊥ Institute of Chemistry, Federal University of Gioias, Goiania, GO 74690-900, Brazil; ∇ Institute of Exact Sciences and Nature, University of International Integration of Afro-Brazilian Lusophony, Redenção, CE 62790970, Brazil; # Research Center on Neglected Diseases, University of Guarulhos (NPDN-UNG), Guarulhos, SP 07030-010, Brazil; ∫ Research Center on Neglected Diseases, University Brazil (NPDN-UB), São Paulo, SP 05508-070, Brazil; 9 Nucleus of Bioassays, Biosynthesis and Ecophysiology of Natural Products (NuBBE), Department of Organic Chemistry, Institute of Chemistry, São Paulo State University (UNESP), P.O. Box 355, Araraquara, SP 14800-900, Brazil

## Abstract

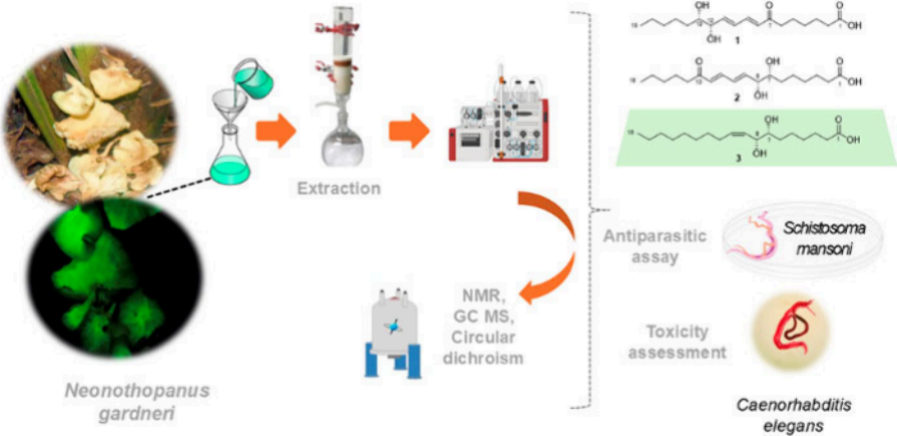

With praziquantel being the sole available drug for schistosomiasis, identifying novel anthelmintic agents is imperative. A chemical investigation of the fruiting body of the bioluminescent mushroom *Neonothopanus gardneri* Berk. resulted in the isolation of new conjugated long-chain fatty acids (8*E*,10*E*,12*S*,13*S*)-12,13-dihydroxy-7-oxo-octadeca-8,10-dienoic acid (**1**) and (7*S*,8*S*,9*E*,11*E*)-7,8-dihydroxy-13-oxo-octadeca-9,11-dienoic acid (**2**) and three previously described compounds, (7*R*,8*R*,9*Z*)-7,8-dihydroxyoctadec-9-enoic acid (**3**), (2*E*)-dec-2-ene-1,10-dioic acid (**4**), and a ketolactone marasmeno-1,15-dione (**5**). Their planar structures were elucidated based on 1D and 2D NMR and MS/MS spectroscopic analyses. Compound **3** displayed significant antiparasitic activity against *Schistosoma mansoni ex vivo* (EC_50_ < 10 μM). No toxicity was observed in mammalian cells or *Caenorhabditis elegans*.

Schistosomiasis, a neglected tropical disease caused by infection with parasitic blood flukes of the genus *Schistosoma*, affects nearly 250 million people worldwide. This disease is intricately linked with poverty and inflicts severe debilitation, leading to chronic ill health.^1^ Despite the extensive use of praziquantel as the primary anthelmintic drug over four decades, the urgency for new therapeutic interventions remains paramount, underscored by the World Health Organization’s ambitious goal to eliminate schistosomiasis as a public health concern by 2030.^2^

Natural products present a promising avenue for addressing schistosomiasis due to their diverse chemical compositions and mechanisms of action. These serve as a potential source of new drug prototypes that may overcome drug resistance. Recent investigations into various natural products have unveiled their promising antischistosomal activity.^2−5^ Moreover, harnessing natural products aligns seamlessly with the principles of sustainable and integrated strategies for neglected tropical diseases.^6,7^

Among the various natural resources currently being studied, the bioluminescent mushroom *Neonothopanus gardneri*, known as Coconut Flower, belonging to the Marasmiaceae family,^8^ has emerged as a promising source for obtaining secondary metabolites with biological potential. In particular, *N. gardneri* displays an intense luminescence yellow from its mycelium and basidiomes in dark environments.^9^ Although the bioluminescent mechanisms have not been fully unraveled, recent discoveries have revealed several bioluminescent molecules, including 3-hydroxyhispidine in *N. nambi* and *Panellus stipticus* and riboflavin in *Mycena chlorophos*.^9^

There are no reports in the literature on the secondary metabolites obtained from the bioluminescent mushroom *N. gardneri*, and the only investigations into this species are focused on unravelling its bioluminescent mechanism.^10^ Therefore, our efforts in this article explore the novel chemical constituents obtained from *N. gardneri* and investigate their antiparasitic and antifungal activities. The extracts of the bioluminescent mushroom *N. gardneri* were subjected to solvent fractionation, C_18_ flash chromatography (ODS), and reversed-phase high-performance liquid chromatography (HPLC) to obtain two new compounds (**1** and **2**) and three compounds previously described in the literature (**3**–**5**, Figure 1). The new structures were elucidated by extensive nuclear magnetic resonance (NMR) spectroscopic analysis, MS/MS spectroscopic analyses, and density functional theory (DFT) analysis. All the compounds described in the article were tested on *Schistosoma mansoni.* Compound **3** had no toxicity to mammalian cells or *Caenorhabditis elegans*, but showed significant antiparasitic activity against *S. mansoni ex vivo* (EC_50_ < 10 μM).
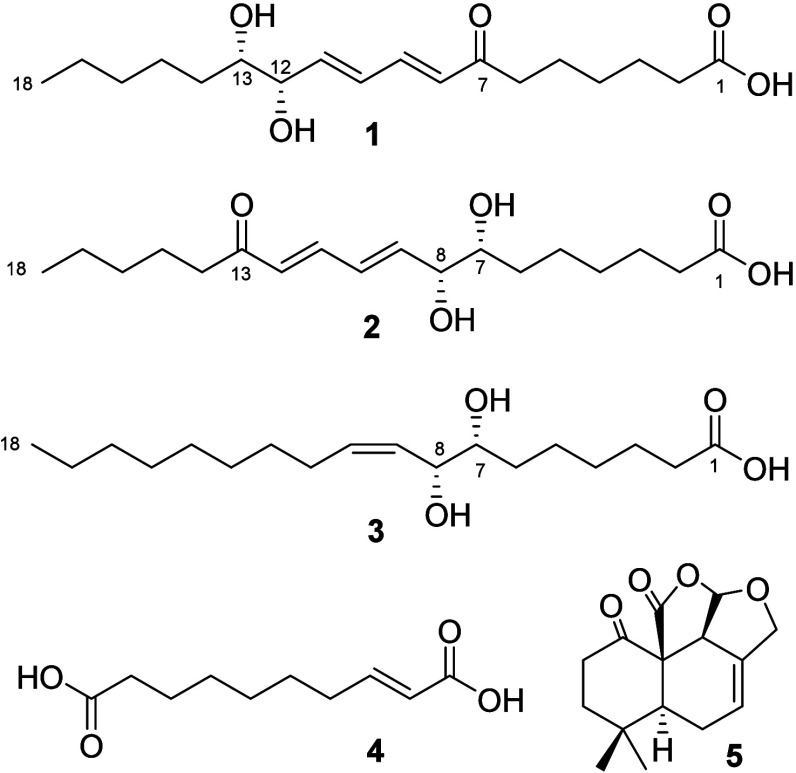


**Figure 1:**
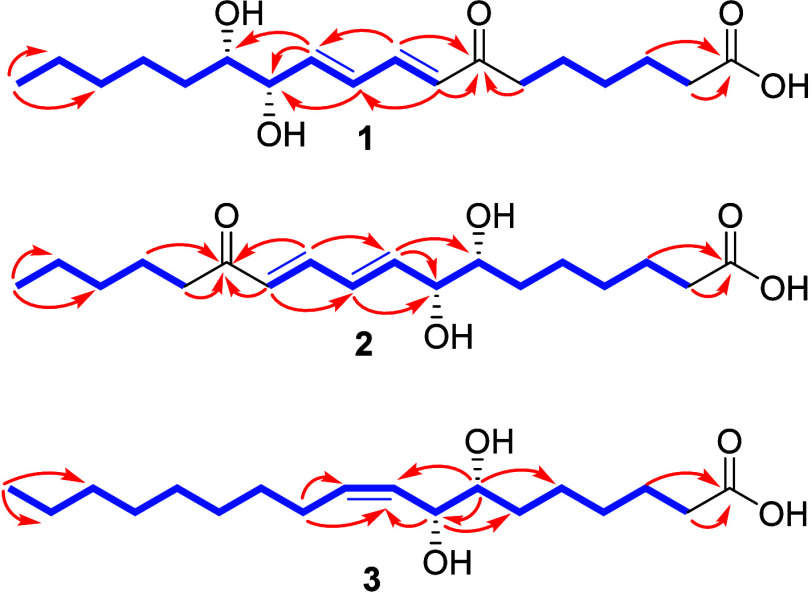
COSY (blue) and HMBC (red) correlations of compounds **1**–**3**.

## Results and Discussion

(8*E*,10*E*,12*S*,13*S*)-12,13-Dihydroxy-7-oxo-octadeca-8,10-dienoic acid (**1**) was obtained as a light-yellow oil. Its molecular formula, C_18_H_31_O_5_, was determined from high-resolution electrospray ionization mass spectrometry (HRESIMS) data, indicating four degrees of unsaturation. Absorption bands of OH (3445 cm^–1^), C=O (1708 and 1598 cm^–1^), and C–H of sp^3^ carbons (2921 and 2852 cm^–1^) were observed in the IR spectrum. The maximum UV absorption at 273 (log ε 2.43) nm indicated the presence of an α,β-unsaturated enone.^12^

The ^1^H NMR data (Table 1) and ^1^H–^1^H correlation spectroscopy (COSY) spectra of **1** showed signals due to two sets of hydrogens on *trans*-olefinic carbons coupled with each other at δ_H_ 6.21 (1H, d, 15.6 Hz, H-8), δ_H_ 7.29 (1H, dd, 15.6, 10.9 Hz, H-9), δ_H_ 6.49 (1H, ddd, 15.3, 10.9, 1.3 Hz, H-10), and δ_H_ 6.31(1H, dd 15.3, 5.9 Hz, H-11), with the coupling constant (*J*) being between 12 and 18 Hz, indicating that the bonds are *trans.* The ^1^H NMR data also showed a triple doublet at δ_H_ 4.08 (dt, 5.9, 1.3 Hz, H-12) and a double doublet at δ_H_ 3.49 (1H, ddd, 8.9, 5.9, 3.3 Hz, H-13) attributed to the carbinolic hydrogens. In addition to the general data for carbinolics and olefins, the ^1^H NMR spectrum showed a methylenic hydrogen signal at δ_H_ 2.28 (t, 7.4 Hz) and δ_H_ 2.63 (t, 7.3 Hz), characteristic of a low-shielding environment. It also revealed multiplets in intense individual absorptions of methylenic groups at δ_H_ 0.91, 1.35, 1.33, and 1.40, which are compatible with linear chain hydrogens.

**Table 1:** ^1^H and ^13^C NMR Data of Compounds **1**–**3** in CD_3_OD

	**1**			**2**			**3**		
position	δ_C_, type	δ_H_ (*J* in Hz)	*g*HMBC	δ_C,_ type	δ_H_ (*J* in Hz)	*g*HMBC	δ_C,_ type	δ_H_ (*J* in Hz)	*g*HMBC
1	177.9, C	-		177.9, C	-		177.9, C	-	
2	35.1, CH_2_	2.28, t (7.4)	1, 3, 4	35.1, CH_2_	2.28, t (7.4)	1, 3, 4	35.2, CH_2_	2.28, t (7.4)	1, 3, 4
3	26.1, CH_2_	1.62, m	1, 2, 4	26.2, CH_2_	1.61, m	1, 2, 4	26.2, CH_2_	1.60, m	1, 2, 4
4	30.2, CH_2_	1.35, m		30.3, CH_2_	1.34, m		30.6, CH_2_	1.34, m	
5	25.5, CH_2_	1.62, m		26.8, CH_2_	1.34, m		26.8, CH_2_	1.60, m	
6	41.1, CH_2_	2.63, t (7.3)	7	33.6, CH_2_	1.40, m/1.61, m		33.8, CH_2_	1.39, m/1.53, m	
7	203.8, C	-		75.4, CH	3.49, tt (5.4, 1.8)		76.1, CH	3.36, ddd (9.0, 6.6, 2.8)	5, 6, 8, 9
8	130.6, CH	6.21, d (15.6)	7, 10	76.1, CH	4.08, dt (5.4, 1.4)	7, 9, 10	72.0, CH	4.20, dd (9.0, 6.6)	6, 7, 9, 10
9	144.3, CH	7.29, dd (15.6, 10.9)	7, 11	145.4, CH	6.31, dd (15.3, 5.4)	7, 8, 11	130.5, CH	5.37, ddt (11.0, 9.0, 1.5)	11
10	130.6, CH	6.49, ddd, (15.3, 10.9, 1.3)	9, 12, 13	130.6, CH	6.49, ddd (15.3, 10.9, 1.4)	8, 11, 12	134.7, CH	5.57, dt (11.0, 7.8)	8, 11
11	145.4, CH	6.31, dd (15.3, 5.9)	9, 12, 13	144.3, CH	7.28, dd (15.5, 10.9)	9, 13	29.1, CH_2_	2.13, ddd (15.6, 7.8, 1.5)	9, 10
12	76.2, CH	4.08, dt (5.9, 1.3)		130.6, CH	6.21, d (15.5)	10, 13	30.4, CH_2_	1.39, m	
13	75.6, CH	3.49, ddd (8.9, 5.9, 3.3)		204.0, C	-		30.9, CH_2_	1.34, m	
14	33.5, CH_2_	1.40, m, 1.55, m		41.2, CH_2_	2.61, t (7.4)	13, 15, 16	30.8, CH_2_	1.34, m	
15	26.2, CH_2_	1.33, m		25.4, CH_2_	1.61, m	13	30.6, CH_2_	1.34, m	
16	33.5, CH_2_	1.33, m		32.7, CH_2_	1.34, m		33.2, CH_2_	1.34, m	
17	23.9, CH_2_	1.35, m	18	23.7, CH_2_	1.34, m	18	23.9, CH_2_	1.34, m	18
18	14.6, CH_3_	0.91, t (7.2)	16, 17	14.4, CH_3_	0.91, t (7.1)	16, 17	14.6, CH_3_	0.9, t (6.9)	16, 17

The ^13^C NMR data (Table 1) showed a carboxylic acid carbon at δ_C_ 177.9 (C-1), a ketone carbon at δ_C_ 203.8(C-7), four olefinic carbon (δ_C_ 130.6, 144.3, 130.6, and 145.4) to two conjugated *E*,*E*-form enone systems, and two oxygenated methine carbons (δ_C_ 76.2 and 75.6). Based on the NMR data mentioned above and the degree of unsaturation of the structure, compound **1** consists of conjugated long-chain fatty acids.

Analysis of the data from heteronuclear single quantum coherence spectroscopy (HSQC) and heteronuclear multiple bond correlation (HMBC) showed the presence of 16 protonated carbons, 1 methyl, 9 methylenes, 6 methines, and 2 nonprotonated carbons, including one acid carbonyl and one ketone. The correlations in *g*HMBC (Figure 1) showed the CH_2_ group at δ_H_ 2.63 (2H, t, *J* = 7.3 Hz, H-6) correlating with the carbon at δ_C_ 203.4 (C-7), indicating the position of the ketone unit in the molecule. The cross-correlations of the doublet at δ_H_ 6.21 with δ_C_ 203.8 (C-7) and 130.6 (C-10) and the double doublet at δ_H_ 7.29 with C-7 and δ_C_ 144.3 (C-10) (Figure 1) confirmed the doubly conjugated ketone. A correlation of the methyl group at δ_H_ 0.91 with δ_C_ 33.5 (C-16) and δ_C_ 23.9 (C-17) and the multiplet at δ_H_ 1.35 (2H, m, H-17) with the signal to δ_C_ 14.6 (C-18) were also observed in the *g*HMBC of the triplet at δ_H_ 2.28 with δ_C_ 177.9 (C-1), δ_C_ 26.1 (C-3), and δ_C_ 30.2 (C-4), and correlations of the δ_H_ 1.62 multiplet with the δ_C_ 177.9 (C-1), δ_C_ 35.1(C-2), and δ_C_ 30.2 C-4 carbons were also observed, confirming the acid unit of compound **1**.

The positions of the double bonds and the carbinols were confirmed using the 1D-TOCSY experiment through a spin system formed between the hydrogens H-8, H-9, H-10, H-11, H-12, and H-13 from the irradiation in H-10 and a second spin system between H-6, H-5, H-4, H-3, and H-2 when irradiated at H-6, thus confirming the position of the ketone carbonyl on carbon C-7 (Figure S3).

The identity of compound **1** was supported by HRESIMS/MS fragmentation analysis (Figure S3) with the molecular ion peak [M + H]^+^ at *m*/*z* 327.2185. The key fragment ions at *m*/*z* 309.2061 [M + H – H_2_O]^+^ and *m*/*z* 291.1955 [M + H – 2H_2_O]^+^ revealed sequential cleavage and confirmed the compound’s structure.

The conformational analysis of compound **1** was carried with an electronic structure calculation using the pm6 semiempirical method.^11^ For compound **1**, the DFT conformational analysis showed lower energies and enthalpy values for the *S,S* conformer. Thus, compound **1** was determined to be (8*E*,10*E*,12*S*,13*S*)-12,13-dihydroxy-7-oxo-octadeca-8,10-dienoic acid.

(7*S*,8*S*,9*E*,11*E*)-7,8-Dihydroxy-13-oxo-octadeca-9,11-dienoic acid (**2**) showed in the HRESIMS a molecular ion peak of *m*/*z* 327.2163 [M + H]^+^ for the expected molecular formula of C_18_H_31_O_5_, with 4 indices of hydrogen deficiencies, corresponding to two unsaturations and two carbonyl groups, agreeing with ^13^C NMR data. The ^1^H NMR data (Table 1) revealed signals due to two sets of hydrogens on *trans*-olefinic carbons coupled to each other at δ_H_ 6.31 (1H, dd, 15.3, 5.4 Hz, H-9), δ_H_ 6.49 (1H, ddd, 15.3, 10.9, 1.4 Hz, H-10), δ 7.28 (1H, dd, 15.5, 10.9 Hz, H-11), and δ_H_ 6.21 (1H, d, 15.5, Hz, H-12) and showed two carbinolic hydrogen signals at δ_H_ 3.49 (1H, tt, 5.4, 1.8, H-7) and δ_H_ 4.08 (1H, dt, 5.4, 1.4, H-7). The ^13^C NMR and HSQC spectra (Table 1) showed the presence of 16 protonated carbons, 1 methyl, 9 methylenes, 6 methines (δ_C_ 75.4, 76.1, 145.4, 130.6, 144.3, and 130.6), and 2 nonprotonated carbons including one acid carbonyl (δ_C_ 177.9) and one ketone (δ_C_ 204.0), which indicated that **2** was similar to **1**.

The *g*HMBC (Figure 1) evidenced correlations with the hydrogen (2H-14) signals at δ_H_ 2,61 with carbons C-13, C-15, and C-16, correlations of the signal at δ_H_ 4.08 (H-8) with C-7, C-9, and C-10, correlations between the hydrogen signal at δ_H_ 6.31 (H-9) and C-7, C-8, and C-12, correlations between the H-11 (δ_H_ 7.28) and C-9 and C-13, and of the doublet at δ_H_ 6.21 (H-12) with carbons C-10 and C-13, determining the position of the olefinic, carbinolic, and ketone carbonyl bonds.

In addition, a 1D-TOCSY experiment was carried out (Figure S7) in which the irradiation of hydrogen H-12 showed a spin system between H-12, H-11, H-10, H-9, and H-8 and a second spin system between H-14, H-15, H-16, H-17, and H-18. This system showed the ketone carbonyl at C-13 and the olefinic bonds at C-12, C-11, C-10, and C-9, revealing compound **2** as an analogue of compound **1**.

For compound **2,** the DFT conformational analysis indicated that conformer *S,S* is the most stable conformation. Thus, compound **2** was determined to be (7S,8*S*,9*E*,11*E*)-7,8-dihydroxy-13-oxo-octadeca-9,11-dienoic acid.

Compound **3** was determined to be C_18_H_34_O_4_ based on its HRESIMS and NMR data analysis. The IR spectrum of **3** showed the presence of hydroxy units (3400 cm^–1^), carbonyl (1707 cm^–1^), and sp^3^ C–H (2952 and 28521 cm^–1^) groups. The ^1^H NMR spectral data exhibited six protons attributed to a methyl at δ_H_ 0.92, two carbinolic hydrogens at δ_H_ 3.36 and 4.20, two resonances at δ_H_ 5.37 and δ_H_ 5.57 assigned to sp^2^ protons, a triplet at δ_H_ 2.28 (alpha carbonyl), and a doublet of doublets of doublets at δ_H_ 2.13. The ^13^C NMR spectral data showed the presence of two unsaturated carbons at δ_C_ 134.7 and 130.5, including one carbonyl group at δ_C_ 177.9 and two carbinolic groups at δ_C_ 76.1 and 72.0.

The *g*HMBC (Figure 1) showed correlations of the triplet at δ_H_ 2.28 and the multiplet at δ_H_ 1.60 with the carbonyl at δ_C_ 177.9 (C-1). Correlations were also observed for δ_H_ 2.13 with δ_C_ 130.5 (C-9) and δ_C_ 134.7 (C-10); additionally, the double double doublet at δ_H_ 4.20, correlating with δ_C_ 33.8 (C-6), δ_C_ 76.1 (C-7), δ_C_ 130.5 (C-9), and 134.7 (C-10), defined the position of the olefinic bond and the *α,β* position of the carbinolics. Correlations were observed for the double doublet of doublets at δ_H_ 3.36 with δ_C_ 26.8 (C-5), δ_C_ 33.8 (C-6), δ_C_ 72.0 (C-8), and δ_C_ 130.5 (C-9), corroborating the sequence of carbinolics at C7, C8, and the double bond at C9/C10. In addition, *g*HMBC correlations were observed between the broad doublet at δ_H_ 5.57 and the methylene carbon at δ_C_ 72.0 (C-8) and the methine carbon at 29.1 (C-11), the δ_H_ 1.34, and the carbon at δ_C_ 14.6 (C-18). It was attributed to the correlations of the triplet at δ_H_ 0.9 with 33.2 (C-16) and 23.9 (C-17).

The *g*COSY (Figure 1) of compound **3** exhibited correlations between H-2 and H-3 and between H-6 and H-8. A correlation was also observed between H-9/H-10 and H-11.

The 1D-TOCSY experiment of compound **3** interactions was observed among H-2, H-3, H-4, H-5, H-6, and H-7 when irradiated at H-2. Interactions were also observed between H-5, H-6, H-7, H-8, H-9, H-10, H-11, and H-12 when irradiated at H-8, confirming only one spin system for compound **3**, which was supported by *g*HMBC and *g*COSY correlations, confirming the positions of the double bonds and carbinols. The DFT conformational analysis showed lower energies and enthalpy values for the *S,S* conformer, thus suggesting the proposed structure of compound **3** to be 7*S*,8*S*-dihydroxy-9*Z*-octadecenoic acid.

The 7*S*,8*S*-dihydroxy-9*Z*-octadecenoic acid was described as a synthesis product.^12^ This is the first evidence of a compound obtained from natural products and applied to schistosomicidal activity.

The conformational analysis of compounds **1**–**3** was performed using the DFT (B3LYP/6-311+G(d,p)) methodology. The method identified the most stable conformations, which are significantly populated at room temperature using the relative energies, Gibbs free energies, enthalpy, and zero point vibrational energy (ZPE) data (Table S1 of the Supporting Information (SI)).^13^ Compound **1** yielded four conformations with Gibbs free energy lying within the 0–0.6 kcal/mol range, indicating that the conformer *S,S* is the most stable conformation for compound **1**. Compound **2** yielded four conformations with Gibbs free energy lying within the 0–1.3 kcal/mol range, indicating that the conformer *S,S* is the most stable conformation for compound **2**. The calculations of the Gibbs free energy and relative energy of all four conformations (Table S2 of the SI) of compound **3** demonstrated that the conformer *S,S* is one of those with the lowest energy conformation. The other two conformations are 1.4 kcal/mol higher in energy. Compounds **1**–**3** provided four conformations due to the hydroxy substituent, whose DFT conformational analysis displayed lower energies and enthalpy values for the *S,S* conformer. In summary, compounds **1**–**3** exhibit minor structural differences in the carbon chain, and conformational analysis revealed that they correspond to the *S,S* conformers.

Compounds **4** and **5** were identified by comparing UV, IR, ^1^H and ^13^C NMR, HRESIMS, and literature data. Based on this information, it was concluded that compound **4** is (2*E*)-dec-2-ene-1,10-dioic acid, which was previously isolated from the fungus.^14,15^ Tetracyclic [7,5,1,0^1,6^0^12,15^]-5.5-dimethyl-11-oxa-2-oxo-pentadec-8-en-14(13)-lactone (**5**) or marasmeno-1,15-dione was identified in comparison with literature data.^16^

### Efficacy against *S. mansoni* and Toxicity Assessment

The *in vitro* antischistosomal potential of compounds **1**–**5** was evaluated at various concentrations on *S. mansoni* (male and female) to determine their effective concentrations at 50% (EC_50_). Praziquantel (PZQ) was employed as a positive control, while vehicle-treated parasites (0.5% DMSO) were a negative control. Compound **3** exhibited significant antiparasitic effects, demonstrating EC_50_ values of 8.3 and 32.6 μM for male and female schistosomes, respectively (Table 2). In contrast, compounds **1**, **2**, **4**, and **5** (>50.0 μM) showed no activity against *S. mansoni*. PZQ displayed EC_50_ values below 2 μM against adult schistosomes (Table 2).

**Table 2:** *In Vitro* Antischistosomal and Cytotoxic Activities of Compounds **1**–**5** and Praziquantel at 72 h

Group	*S. mansoni* male EC_50_	*S. mansoni* female EC_50_	Vero cell CC_50_	SI^a^ (male worms)	SI^a^ (female worms)
**1**	>50 μM	>50 μM	ND	ND	ND
**2**	>50 μM	>50 μM	ND	ND	ND
**3**	8.3 ± 2.4 μM	32.6 ± 5.1 μM	>200 μM	>24.09	>6.13
**4**	>50 μM	>50 μM	ND	ND	ND
**5**	>50 μM	>50 μM	ND	ND	ND
PZQ	0.9 ± 0.1 μM	1.2 ± 0.1 μM	>200 μM	>250	>333

a Selectivity index: SI = CC_50_/EC_50_. Values are means (±SD) of three independent triplicated experiments. Not determined (ND). Praziquantel (PZQ).

The *in vitro* bioassay revealed a concentration-dependent antiparasitic effect, with male worms exhibiting greater susceptibility to compound **3** than females (Figure 1). Although the underlying reasons for this sex-dependent drug sensitivity in schistosomes are not fully understood, it is worth noting that several natural products with antischistosomal properties are known to exert a more substantial effect on male parasites.^17−19^

Since compound **3** belongs to the carboxylic acid group, its antischistosomal effect is consistent with the activities reported for other compounds, such as *ent*-kaurane diterpenes^20^ and oxopopulifolic acids.^21^ Compound **3** exhibited no toxicity against Vero cells, with CC_50_ values exceeding 200 μM, demonstrating a favorable SI > 24 for male schistosomes (Table 2). It did not show toxicity to *C. elegans*, demonstrating its biological potential.

Although ketolactone marasmeno-1,15-dione (**5**) belongs to the highly bioactive class of sesquiterpene lactones, which have shown promise against schistosomes in both *in silico*(22) and *in vitro*(23,24) studies, no antiparasitic activity was detected in this study. The absence of efficacy could be attributed to structural differences or the specific concentrations tested. Future investigations could explore structurally related compounds to identify key factors influencing their effectiveness and selectivity against *S. mansoni*.

These findings align with previous studies demonstrating the low toxicity potential of other carboxylic acid derivatives,^13,25^ further reinforcing the safety profile of **3**. The absence of toxicity in both cellular and *in vivo* models highlights the therapeutic promise. It underscores the potential of this class of compounds for further exploration in antischistosomal drug development.

## Experimental Section

### General Experimental Procedures

UV spectra were recorded on a PDA-HPLC system, and IR spectra were obtained using an Agilent Cary 630 Fourier transform (FTIR) apparatus equipped with a diamond crystal ATR measurement accessory. ^1^H NMR (600 MHz), ^13^C NMR (150 MHz), HMBC, HSQC, and COSY experiments were recorded on a Bruker Avance DRX-600 spectrometer using the residual nondeuterated (CD_3_OD) signal as an internal standard. High-resolution mass spectrometry data were measured using a Thermo Fisher Scientific LTQXL-Discovery Orbitrap instrument coupled to an Accela UPLC-DAD system. The following conditions were used for mass spectrometric analysis: capillary voltage 45 V, capillary temperature 320 °C, auxiliary gas flow rate 10–20 arbitrary units, sheath gas flow rate 40–50 arbitrary units, spray voltage 4.5 kV, mass range 100–2000 amu, resolution 30 000 for MS and 7500 for MS/MS. Preparative and analytical mode HPLC were performed on a Shimadzu, model LC-20 A, UV/vis photodiode-array PDA detector, model SPD-M20 using Phenomenex C_18_ (250 mm × 10.0; mm; 5 μm; 100 Å) and C_18_ Phenomenex Luna (250 × 4.60 mm; 5 μm; 110 Å) columns. Column chromatography (CC) was performed over reversed-phase silica gel, 230–400 mesh (Merck).

### Fungal Material

The mushroom *Neonothopanus gardneri* (Berk. Ex Gardner) was collected in the southwest region in the Mimoso village (S 42.8604, W 6.25258), in the municipality of São Francisco, state of Maranhao, Brazil, in February 2013, between 18 and 20 h (authorization, SISBIO No. 54548-1). The identification of *N. gardneri* was performed by Dr. Cassius Vinicius Stevani of the Laboratory Bioluminescence of Fungi, Institute of Chemistry of the University of São Paulo, Brazil, and a voucher specimen was deposited at the Herbarium of the Institute of Botany of Sao Paulo, Brazil (voucher no. SP 416340).

### Extraction and Isolation

The mushrooms were washed in running water and distilled water and subsequently triturated. The crushed mushrooms (1000 g) were extracted exhaustively with AcOEt for 8 consecutive days. The solvent was evaporated, producing a crude extract of AcOEt (61.7 g, 95%). The AcOEt extract was dissolved in MeCN and subjected to liquid partitioning with hexane. The MeCN fraction was evaporated and produced a yield of 4.22 g, 8.54%. A portion of the CH_3_CN fraction (2.70 g) was fractionated by C_18_ ODS and eluted with an H_2_O–CH_3_OH gradient (70:30 → 0:100), affording eight subfractions (FNg1–FNg8). HPLC purified the subfraction Ng3 (165 mg) (ACN:H_2_O in nonlinear gradient mode 30–100% ACN at 35 min, detection at 254 nm, and flow of 4.5 mL/min), obtaining compounds **1** (*t*_R_ = 12.46 min, 6.1 mg), **2** (*t*_R_ = 14.43 min, 4.1 mg), **4** (*t*_R_ = 7.93 min, 12.7 mg), and **5** (*t*_R_ = 21.15 min, 12.0 mg). HPLC purified the Ng4 subfraction (360 mg) (ACN:H_2_O in nonlinear gradient mode 40–100% ACN at 35 min, detection at 254 nm, and flow of 4.5 mL/min), yielding compound **3** (*t*_R_ = 18.65 min, 8.0 mg).

#### Compound (**1**)

Light yellow oil; UV (MeOH) λ_max_ (log ε) 273 (2.43) nm, Figure S1; IR ATR (MeOH) ν_max_ 3445, 1708, 1598, 2921, and 2852 cm^–1^; ^1^H and ^13^C NMR spectra (Table 1); HRESIMS *m*/*z* [M + H]^+^ calcd for C_18_H_31_O_5_ 327.2166; found 327.2165.

#### Compound (**2**)

Light yellow oil; UV (MeOH) λ_max_ (log ε) 273 (2.43) nm, Figure S2; IR ATR (MeOH) ν_max_ 3449.1731, 1595, 2922, and 2853 cm^–1^; ^1^H and ^13^CNMR spectra, Table 1; HREIMS *m*/*z* [M + H]^+^ calcd for C_18_H_31_O_5_ 327.2166; found 327.2163.

### Computational Details

The DFT calculations were carried out using the GAUSSIAN 03 program. The zero point vibrational energy, relative energies, enthalpy, and Gibbs free energy (kcal/mol) values of the conformers and the Cartesian coordinates for the compounds were obtained using TDDFT at the B3LYP/6-311+G(d,p) level (Table S1 of the Supporting Information (SI)).

### Animal, Parasite, and Cell Maintenance

The *S. mansoni* (BH strain) life cycle was sustained by passage through *Biomphalaria glabrata* snails and Swiss mice at the Research Center on Neglected Diseases (Guarulhos University, SP, Brazil). Vero cells from the American Type Culture Collection (ATCC) were cultured in Dulbecco’s modified Eagle medium (DMEM) supplemented with 10% heat-inactivated fetal calf serum and 2 mM l-glutamine (Vitrocell, Campinas, SP, Brazil). *C. elegans* (strain N2) was maintained at 22 °C on nematode growth medium (NGM) agar seeded with *Escherichia coli* strain OP50, following standard protocols.^26^

### Antiparasitic Assay and Toxicity Assessment

The *in vitro* antischistosomal assay followed established procedures.^27^ Adult schistosomes from infected mice were exposed to test samples (0.78–50 μM) in RPMI 1640 medium supplemented with 5% heat-inactivated fetal calf serum, penicillin (100 U/mL), and streptomycin (100 μg/mL) in a 24-well culture plate. Test samples, including PZQ, were dissolved in DMSO and tested in triplicate with experiments repeated three times. A BEL Engineering microscope assessed parasite viability at 24, 48, and 72 h.

Cytotoxicity was assessed using a previously established method.^20,28^ Cells were plated in 96-well plates and treated with test compounds (6.25–200 μM) and doxorubicin (DXR) at 10 μM as a positive control for 72 h. Cell viability was determined using an MTT solution, and absorbance was measured at 595 nm using a microplate spectrophotometer. The assay was performed in triplicate, and the results are expressed as a percentage of the control.

Toxicity assays with *C. elegans* were performed as previously described.^29^ For 24 h, 26 L4-stage worms were exposed to samples at concentrations ranging from 25 to 200 μM, including levamisole (LVZ) at 10 μM as the positive control. Viability was assessed based on mobility and form using an inverted microscope, with experiments repeated three times.

Statistical analysis was conducted using GraphPad Prism 8.0. CE_50_ and CC_50_ values were determined from sigmoid dose–response curves. Selectivity indices were calculated by dividing CC_50_ values obtained on Vero cells by CC_50_ values determined on *S. mansoni*.^30^

All experiments followed protocols approved by the Committee for the Ethical Use of Animals in Experimentation at Guarulhos University (Guarulhos, SP, Brazil; protocol ID 47/20).

## Supporting Information Available

^1^H and ^13^C NMR spectra of compounds **1**–**5**, infrared and UV–visible spectra, computational details (DFT calculations), and EMIES (PDF)
