# Supplementary material for: Anthelmintic Potential of Conjugated Long-Chain Fatty Acids Isolated from the Bioluminescent Mushroom Neonothopanus gardneri
Source: J Nat Prod. 2025 Jan 4;88(2):255–61. doi: 10.1021/acs.jnatprod.4c00546 (PMC11877520; doi:10.1021/acs.jnatprod.4c00546)
Supplement: Supplementary file 1 — np4c00546_si_001.pdf [file np4c00546_si_001.pdf]

Supporting information of

Anthelmintic properties of lipids and (7Z)-7,8-dihydroxy-octadeca-7-enoic acid from the bioluminescent mushroom *Neonothopanus gardneri*

Maria D. A. Oliveira [†*], Teresinha de Jesus A. dos S. Andrade [‡], Joaquim S. C. Junior [§], Nerilson Marques Lima [┴], Hugo Gontijo Machado [┴], Jioji, N. Tabudravu [‖], Francisco das Chagas Lima Pinto [∇], Lucas Fukui-Silva [#], Monique C. Amaro [#], Josué de Moraes [#∫], Dulce Helena S. Silva [∬], Antônia Maria das Graças L. Citó [†], Chistiane Mendes Feitosa [†·]

[*]*Federal University of Piaui, Department of Chemistry, Campus Ministro Petrônio Portela, Teresina, PI, Brazil.*

[‡]*Nucleus of Applied Research to Sciences-NIAC, Federal Institute of Education, Science and Technology of Maranhao-IFMA, Presidente Dutra (Maranhao)- 65.635-468, Brazil.*

[§]*Federal Institute of Piaui, Department of Chemistry, Campus Central, Praça da Liberdade, Teresina, PI, Brazil.*

[┴]*Chemistry Institute, Federal University of Gioias, 74690-900, Goiania, GO, Brazil.*

[‖]*School of Natural Sciences, Faculty of Science and Technology, University of Central Lancashire PR1 2HE Preston, UK.*

[∇] *Institute of Exact Sciences and Nature, University of International Integration of Afro-Brazilian Lusophony, ZIP 62790970, Redenção, CE, Brazil.*

[#]*Research Center on Neglected Diseases, Guarulhos University (NPDN-UNG), Guarulhos, SP, Brazil.*

[∫]*Research Center on Neglected Diseases, Brazil University (NPDN-UB), São Paulo, SP, Brazil.*

[∬]*Nucleus of Bioassays, Biosynthesis and Ecophysiology of Natural Products (NuBBE), Department of Organic Chemistry, Institute of Chemistry, São Paulo State University (UNESP), P. O. Box 355, 14800-900, Araraquara, SP, Brazil.*

[*,‡] Corresponding authors

[*] Maria D. A. Oliveira, Tel.: +55 86 99905-2578

Email: maralves013@gmail.com

[‡]Teresinha de Jesus A. dos S. Andrade, Tel.: +55 99 98265-5208,

Email: teresinha.andrade@ifma.edu.br

Figure S 1. IR spectrum recorded by of compound (**1**) using liquid solution............................5
Figure S 2. ^{1}H NMR (600 MHz) spectrum of compound (**1**) in CD_3OD..................................6
Figure S 3. ^{13}C NMR (150 MHz) spectrum of compound (**1**) in CD_3OD 6
Figure S 4.^{13}C NMR and DEPT 135 (150 MHz) spectrum of compound (**1**) in CD_3OD..........7
Figure S 5. HSQC spectrum of compound (**1**) in CD_3OD 8
Figure S 6. HMBC spectrum of compound (**1**) in CD_3OD...9
Figure S 7. ^{1}H-^{1}H COSY spectrum of compound (**1**) in CD_3OD 10
Figure S8. ^{1}H-^{1}H TOCSY spectrum of compound (**1**) in CD_3OD..11
Figure S9. HRESIMS spectrum of compound (**1**) 11
Figure S10. UV spectrum (1μg/mL, MeOH) of (**1**)..12
Figure S11. IR spectrum recorded by of compound (**2**) using liquid solution.........................13
Figure S 12. ^{1}H NMR (600 MHz) spectrum of compound (**2**) in CD_3OD..............................14
Figure S 13. ^{13}C NMR (150 MHz) spectrum of compound (**2**) in CD_3OD 15
Figure S 14.^{13}C NMR and DEPT 135 (150 MHz) spectrum of compound (**2**) in CD_3OD.......15
Figure S 15. HSQC spectrum of compound (**2**) in CD_3OD 16
Figure S 16. HMBC spectrum of compound (**2**) in CD_3OD..17
Figure S 17. ^{1}H-^{1}H COSY spectrum of compound (**2**) in CD_3OD 18
Figure S 18. ^{1}H-^{1}H TOCSY spectrum of compound (**2**) in CD_3OD..19
Figure S 19. HRESIMS spectrum of compound (**2**) 19
Figure S 20. UV spectrum (1μg/mL, MeOH) of (**2**)...20
Figure S 21. IR spectrum recorded by of compound (**3**) using liquid solution.........................21
Figure S 22. ^{1}H NMR (600 MHz) spectrum of compound (**3**) in CD_3OD..............................21
Figure S 23. ^{13}C NMR (150 MHz) spectrum of compound (**3**) in CD_3OD 22
Figure S 24.^{13}C NMR and DEPT 135 (150 MHz) spectrum of compound (**3**) in CD_3OD.......22
Figure S 25. HSQC spectrum of compound (**3**) in CD_3OD 23

Figure S 26. HMBC spectrum of compound (**3**) in CD_3OD 24

Figure S 27. ^1H-^1H COSY spectrum of compound (**3**) in CD_3OD 25

Figure S 28. ^1H-^1H TOCSY spectrum of compound (**3**) in CD_3OD 26

Figure S 29. HRESIMS spectrum of compound (**3**) 26

Figure S 30. UV spectrum (1µg/mL, MeOH) of (**3**) 27

List of Supplementary Tables **27**

Table 1: Relative Energies, Enthalpy and Gibbs free energy (kcal/mol).......... 27

Table 2: Relative Energies, Enthalpy and Gibbs free energy (kcal/mol).......... 29

Table 3: Relative Energies, Enthalpy and Gibbs free energy (kcal/mol).......... 31

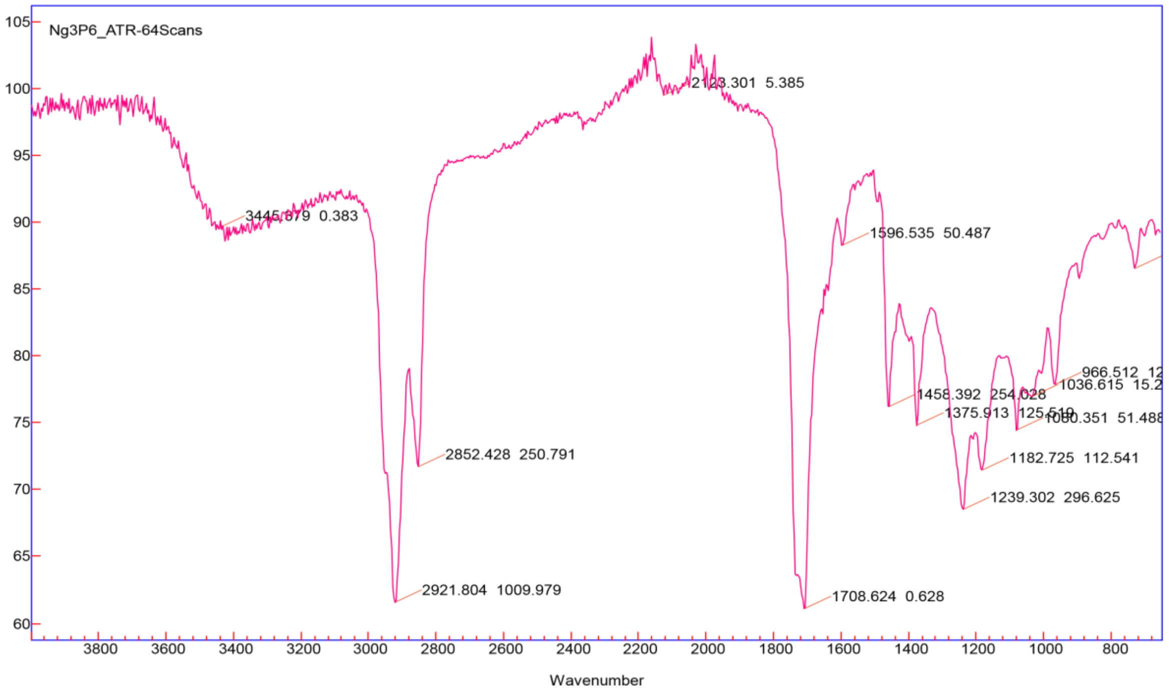

Figure S 1. IR spectrum recorded by of compound (**1**) using liquid solution

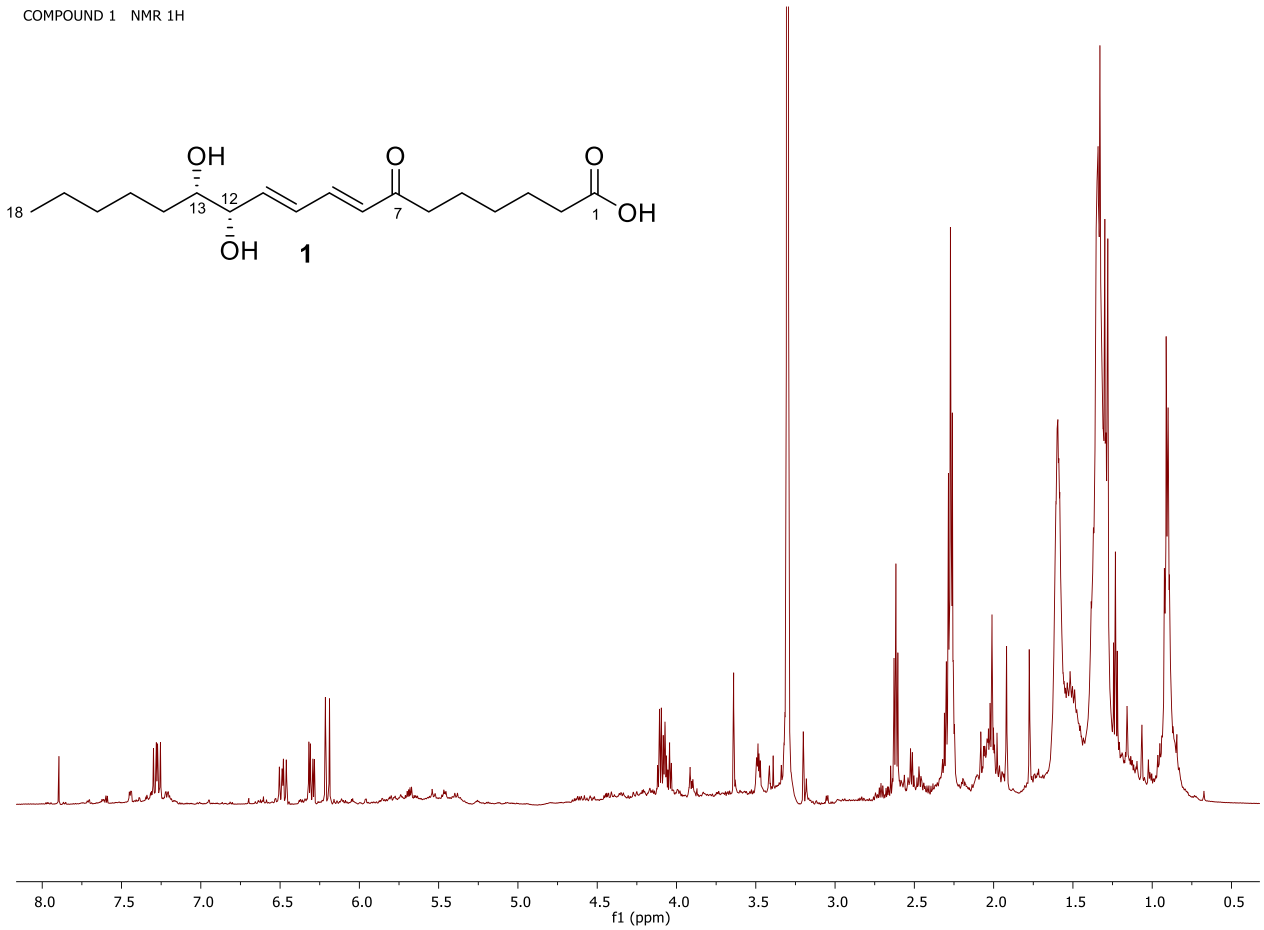

Figure S 2. ^{1}H NMR (600 MHz) spectrum of compound (**1**) in CD_3OD

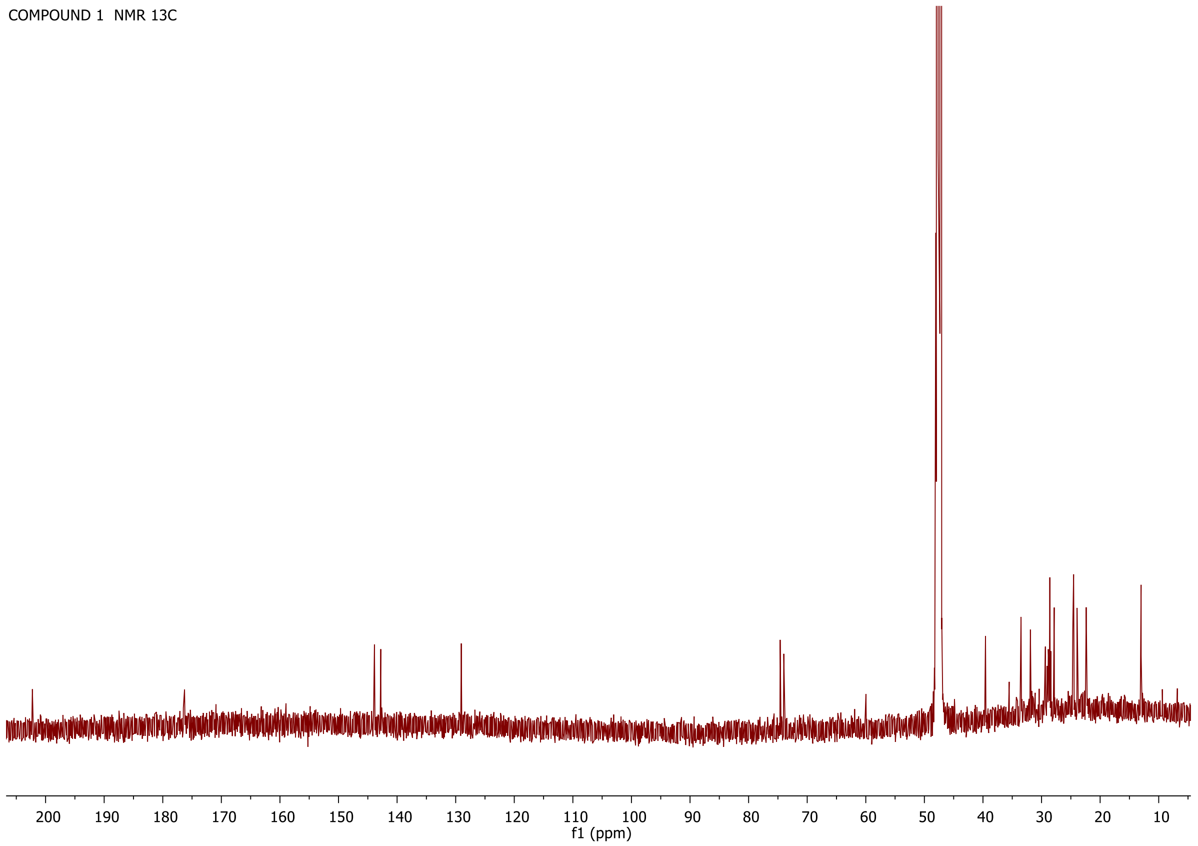

Figure S 3. ^{13}C NMR (150 MHz) spectrum of compound (**1**) in CD_3OD

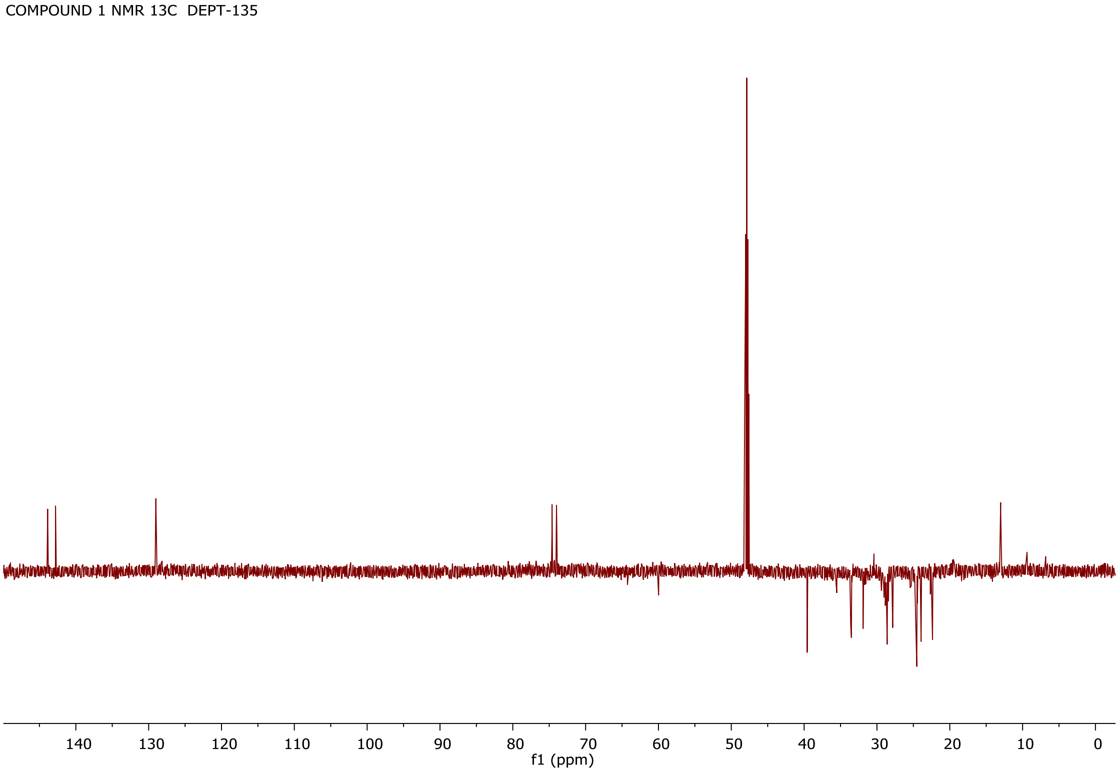

Figure S 4. ^{13}C NMR and DEPT 135 (150 MHz) spectrum of compound (**1**) in CD_3OD

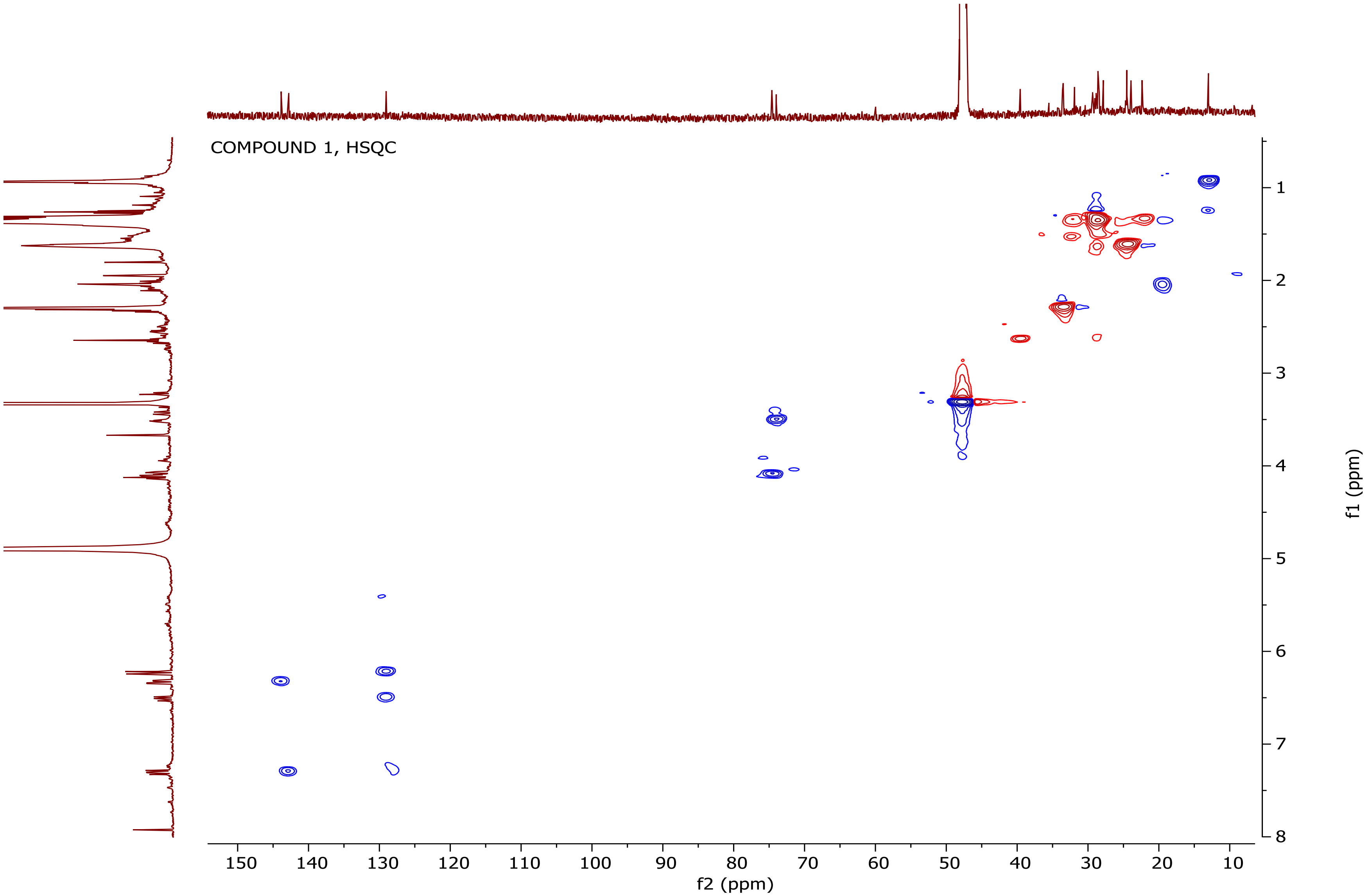

Figure S 5. HSQC spectrum of compound (**1**) in CD_3OD

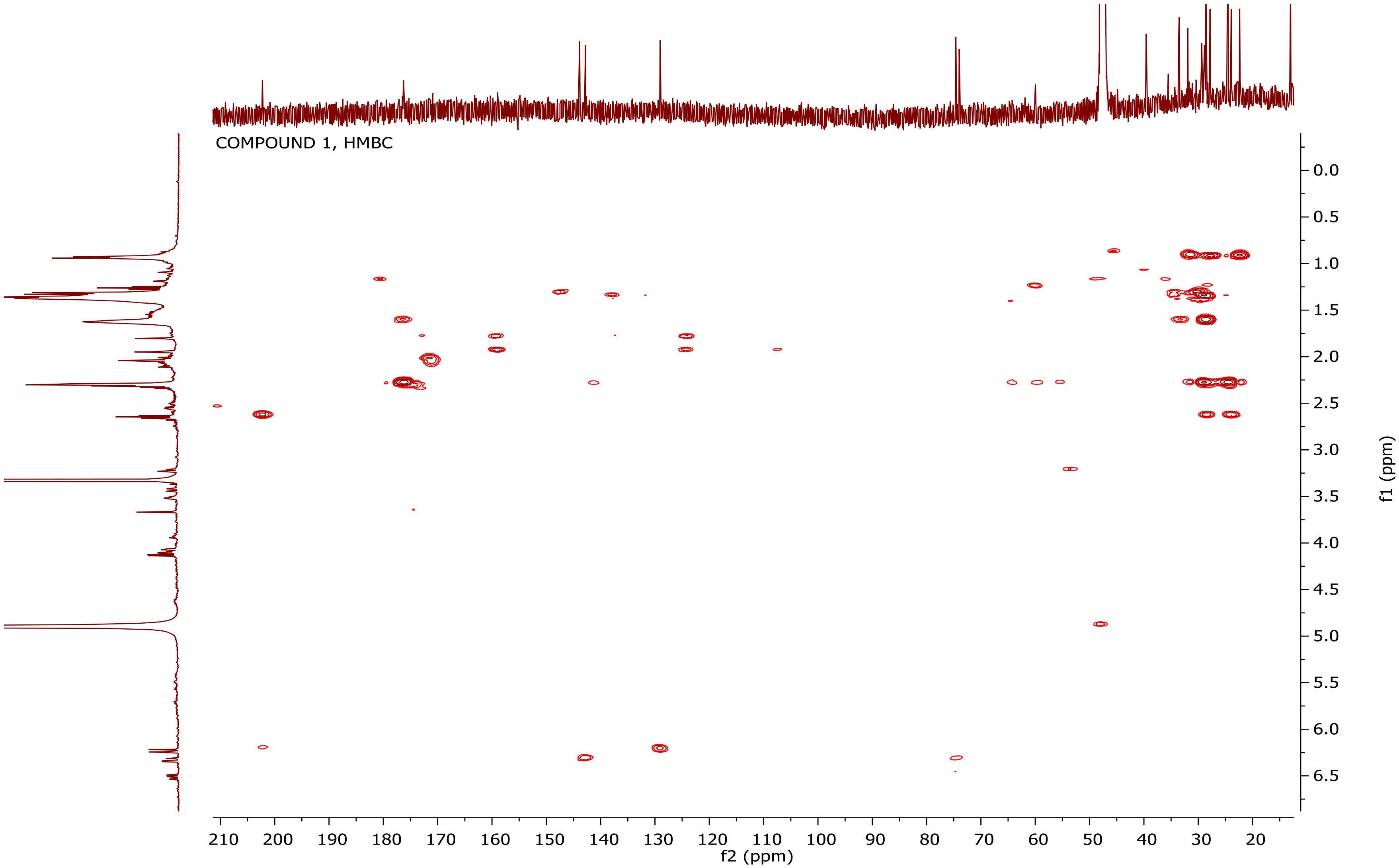

Figure S 6. HMBC spectrum of compound (**1**) in CD_3OD

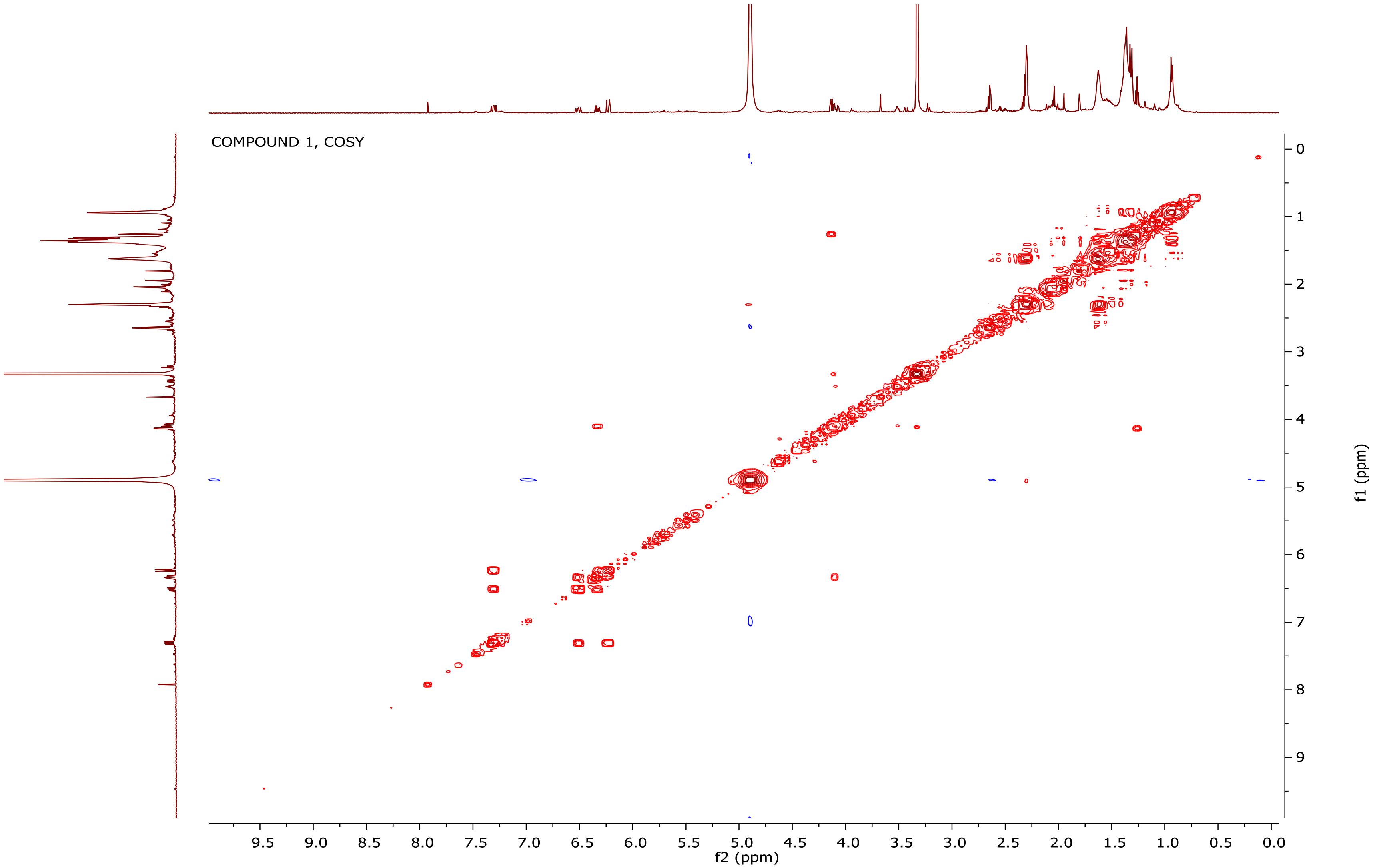

Figure S 7. ^{1}H-^{1}H COSY spectrum of compound (**1**) in CD_3OD

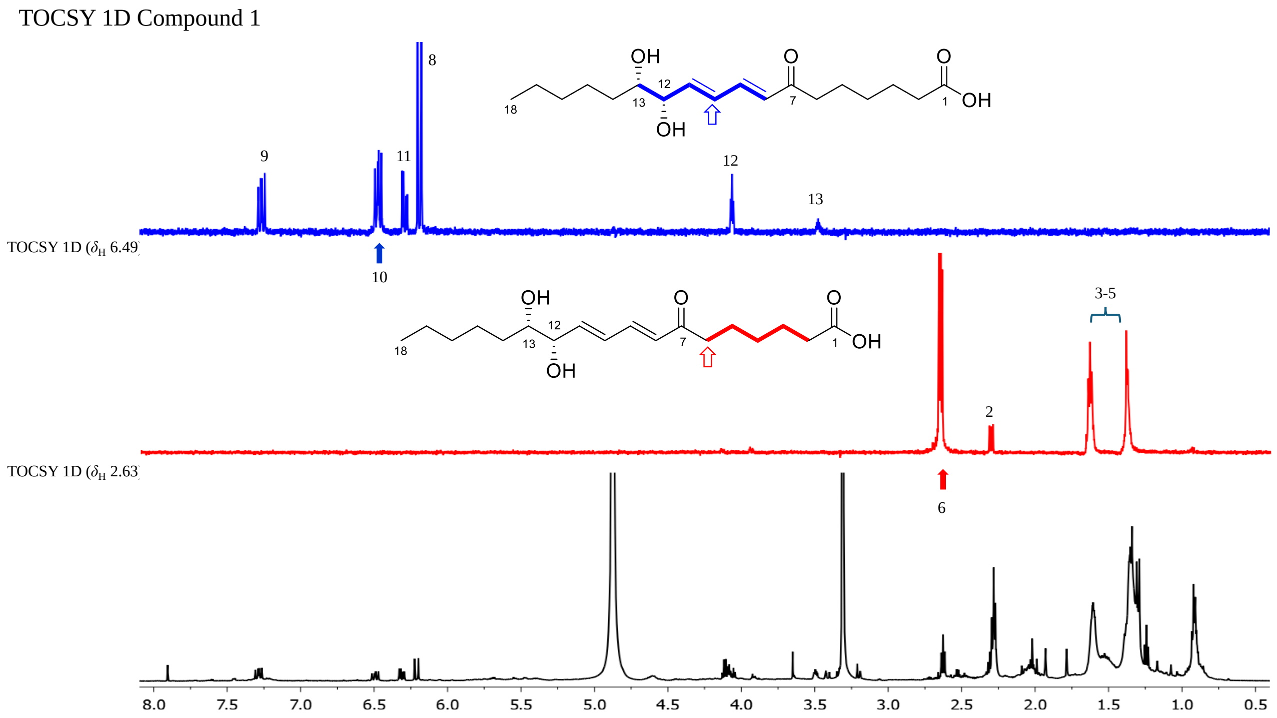

Figure S8. ${}^{1}H$-${}^{1}H$ TOCSY spectrum of compound (**1**) in CD_3OD

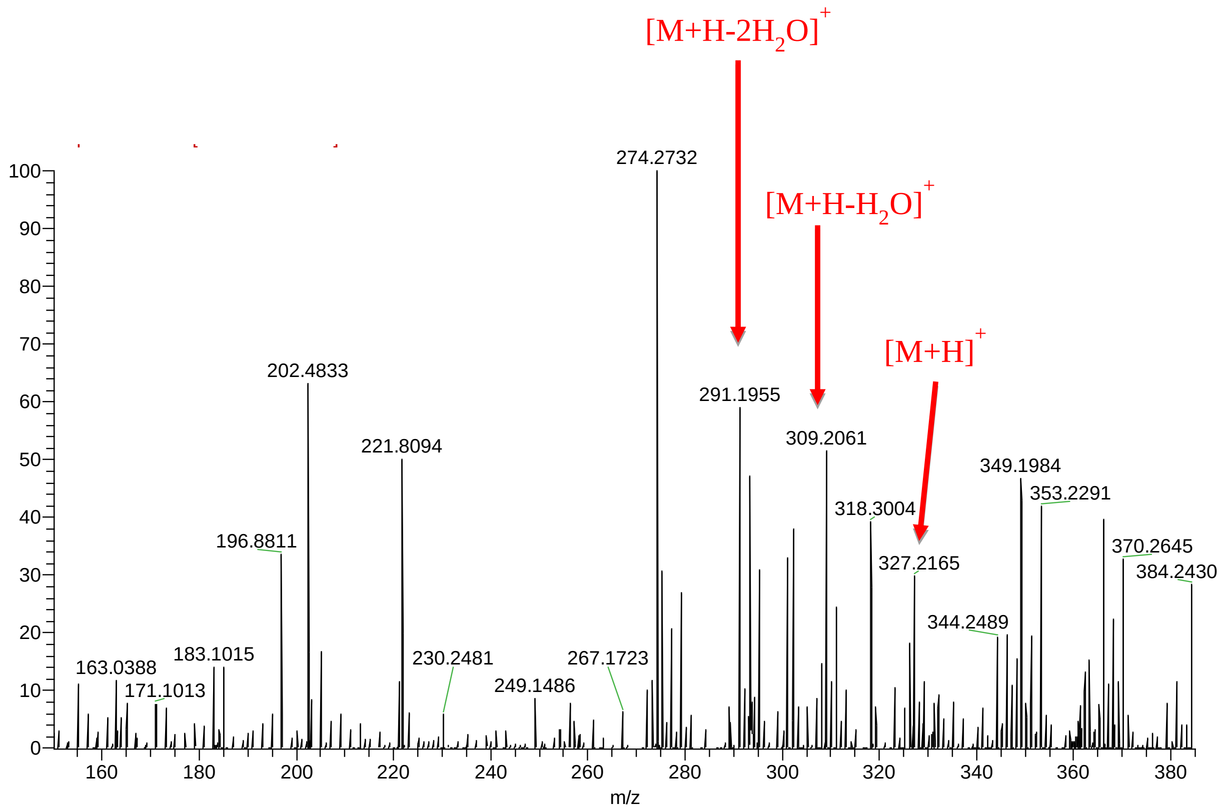

Figure S9. HRESIMS spectrum of compound (**1**)

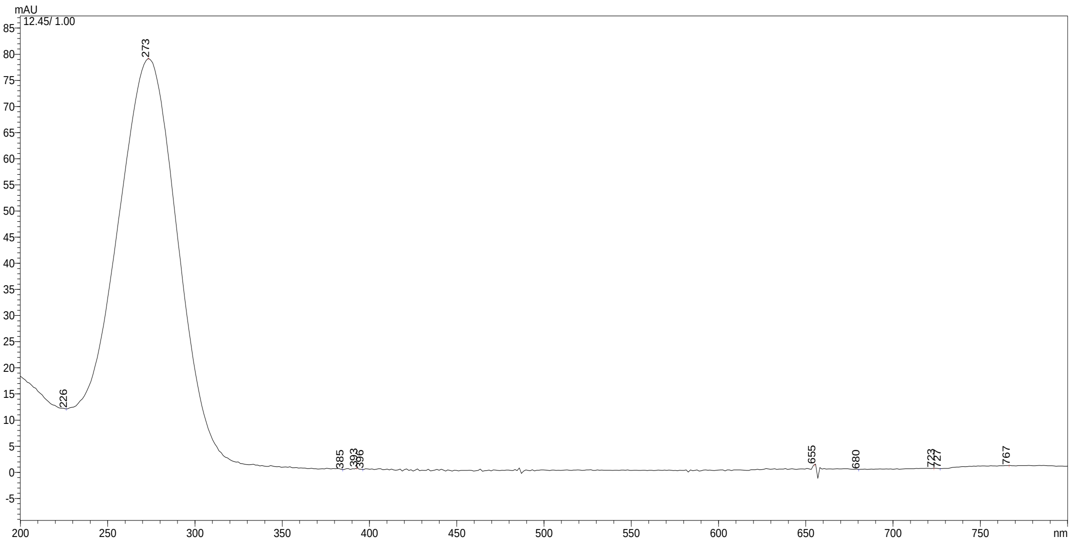

Figure S10. UV spectrum (1µg/mL, MeOH) of (**1**)

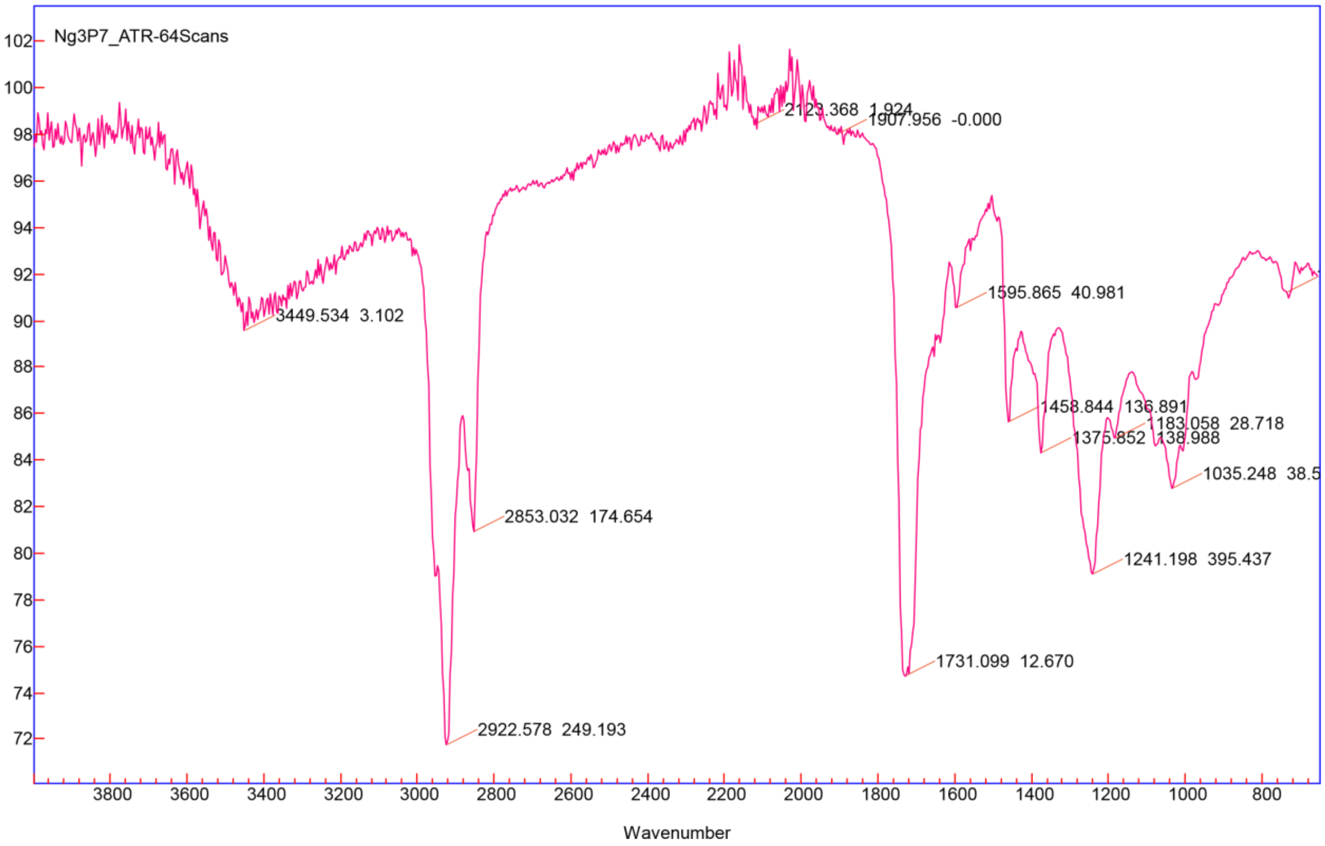

Figure S11. IR spectrum recorded by of compound (**2**) using liquid solution

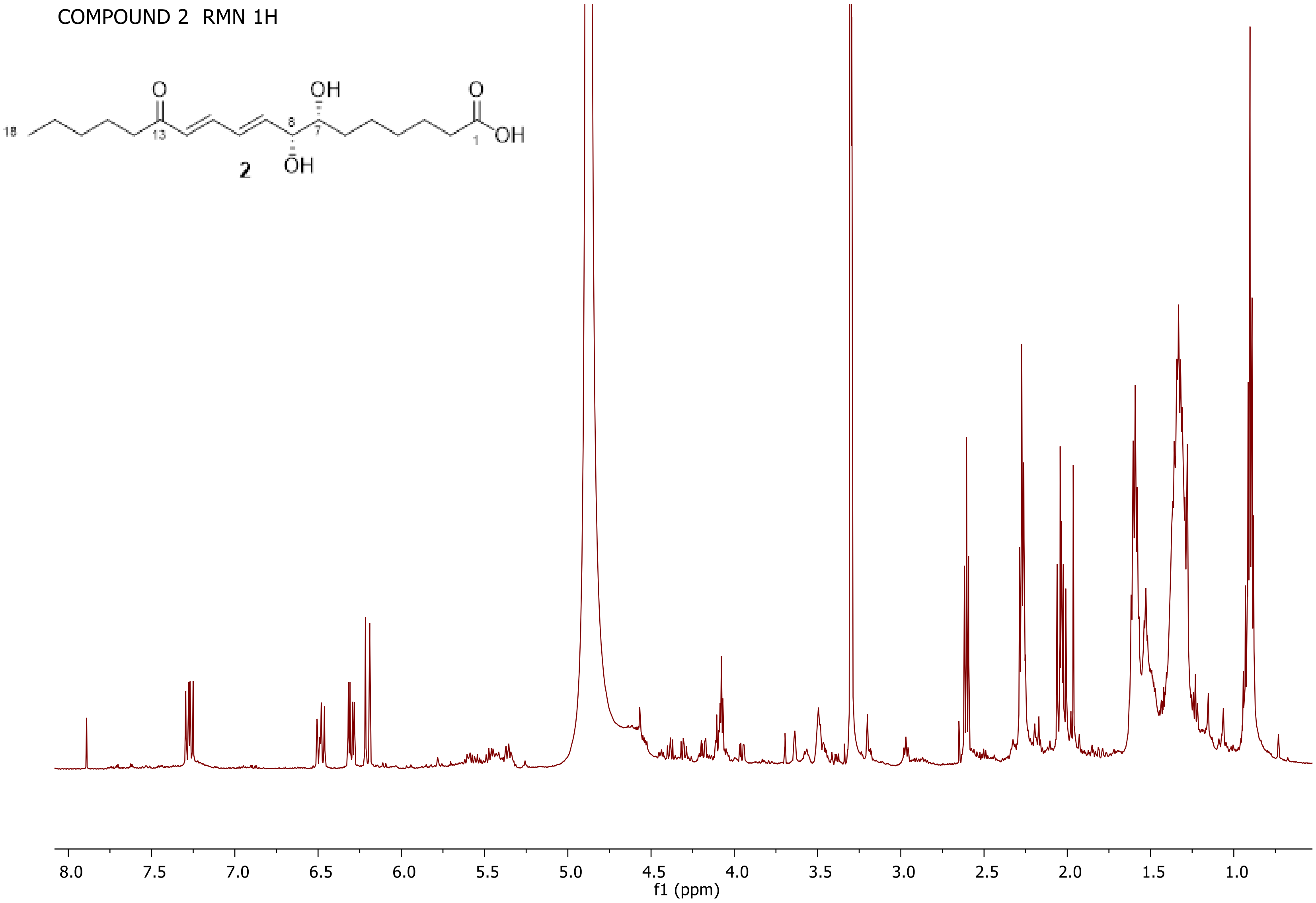

Figure S 12. ^{1}H NMR (600 MHz) spectrum of compound (**2**) in CD_3OD

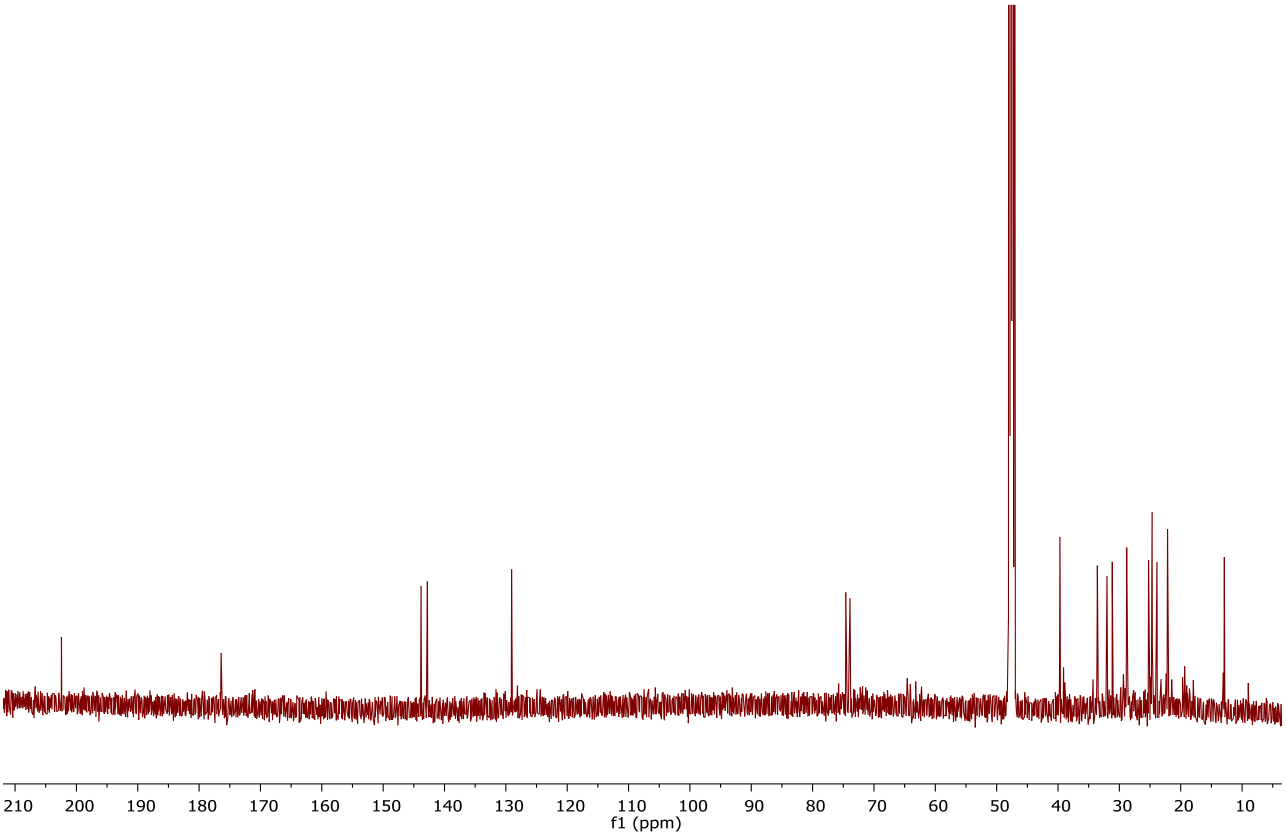

Figure S 13. ^{13}C NMR (150 MHz) spectrum of compound (**2**) in CD_3OD

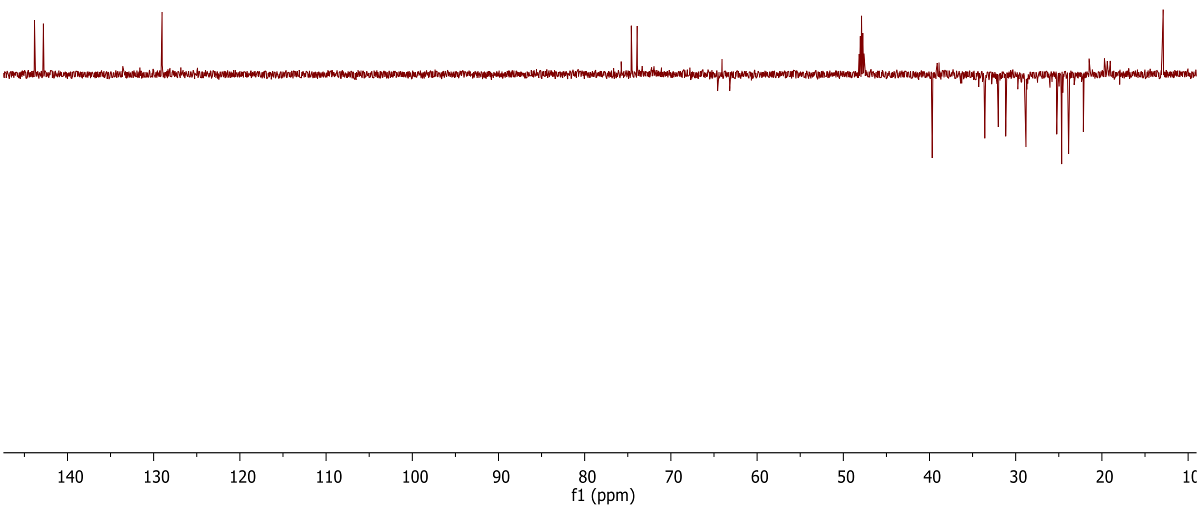

Figure S 14. ^{13}C NMR and DEPT 135 (150 MHz) spectrum of compound (**2**) in CD_3OD

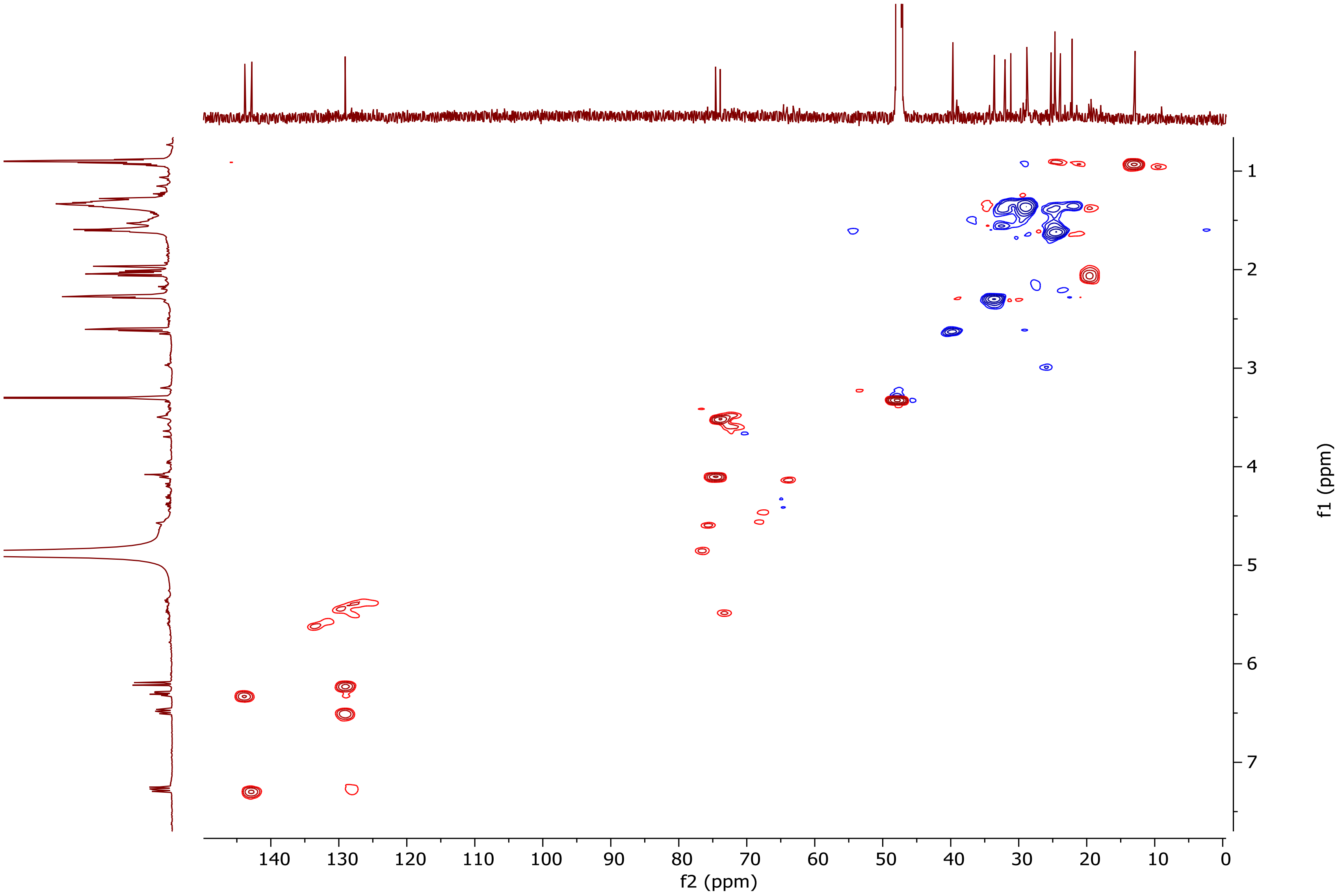

Figure S 15. HSQC spectrum of compound (**2**) in CD_3OD

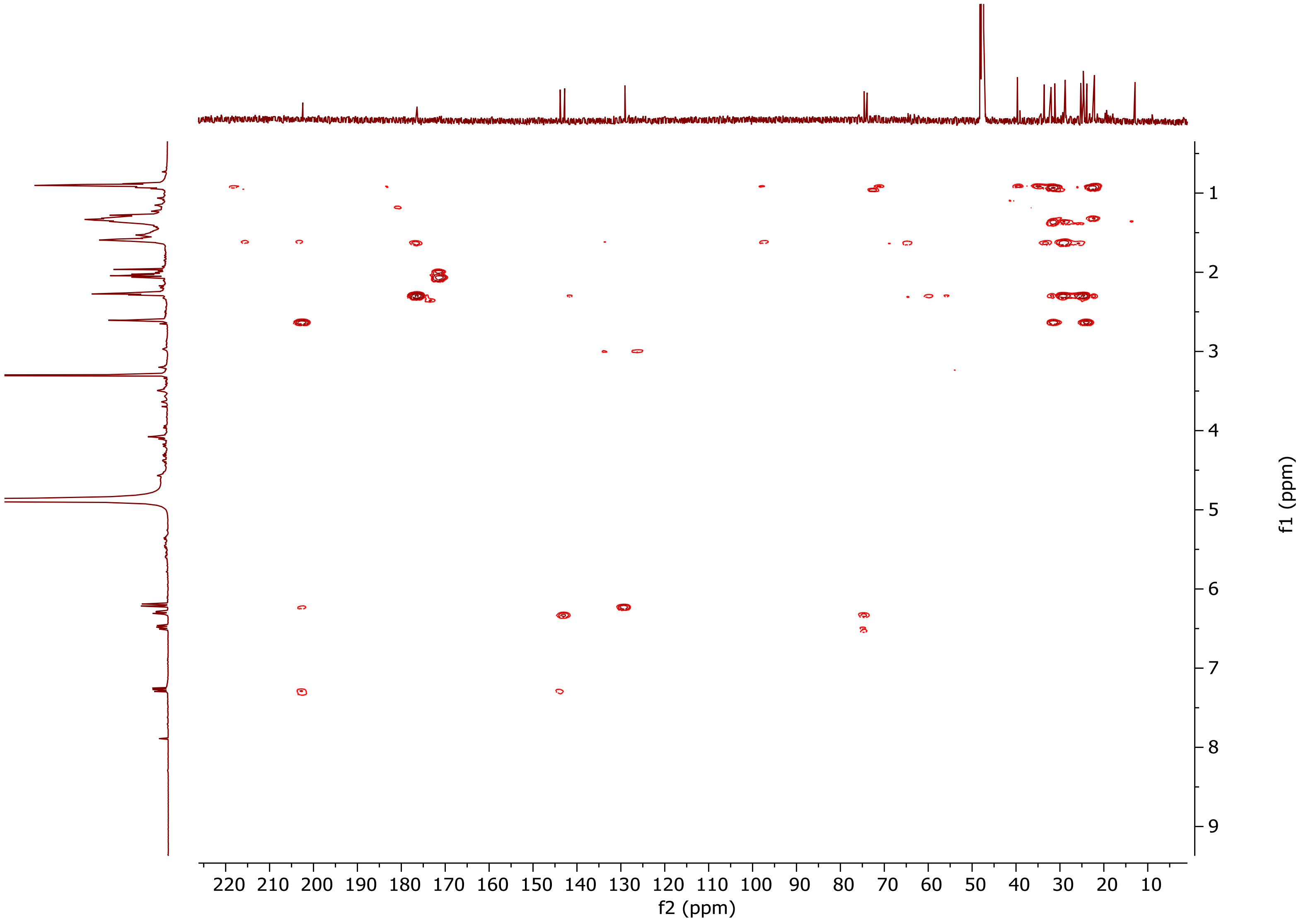

Figure S 16. HMBC spectrum of compound (**2**) in CD_3OD

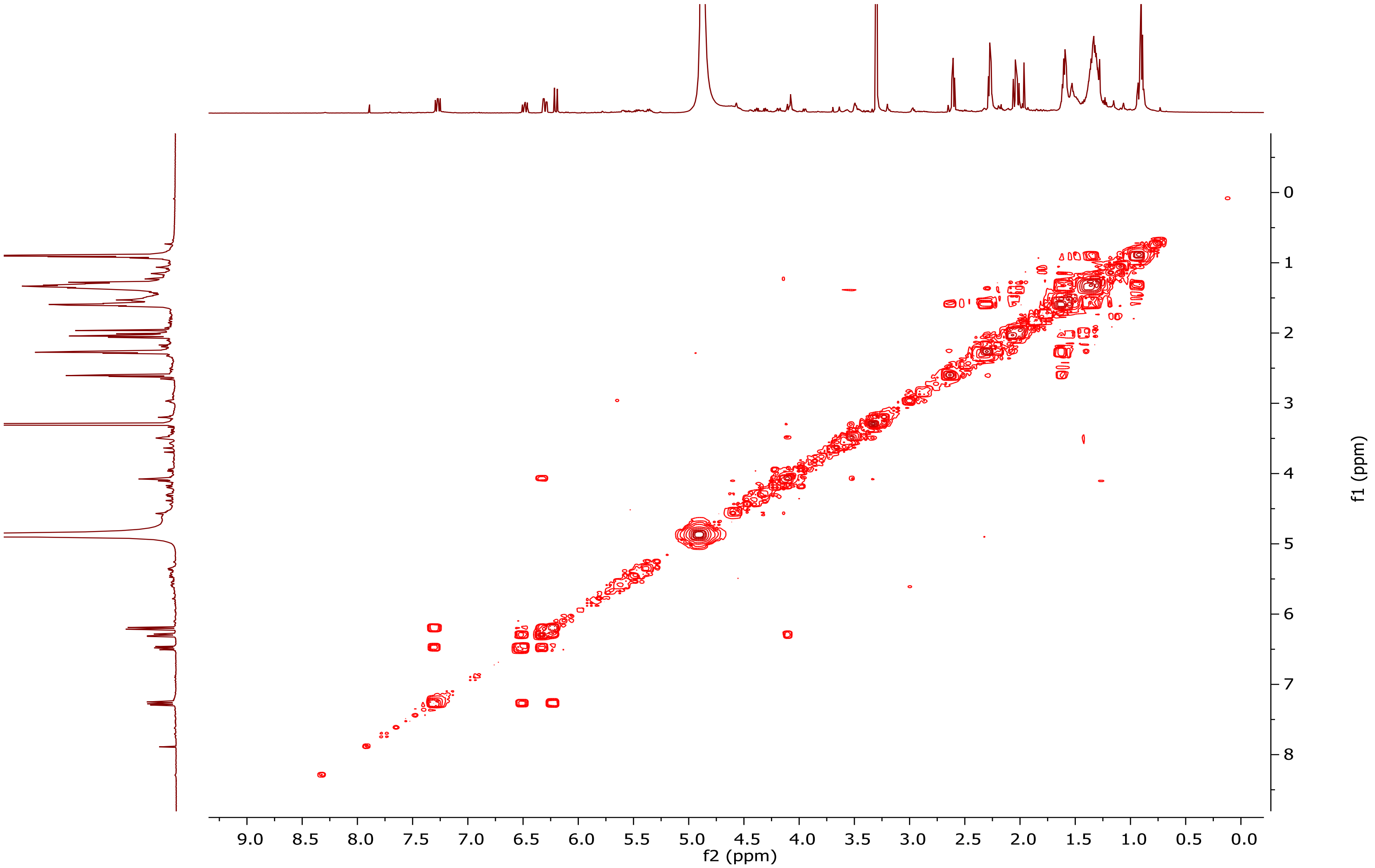

Figure S 17. ^{1}H-^{1}H COSY spectrum of compound (**2**) in CD_3OD

TOCSY 1D Compound 2

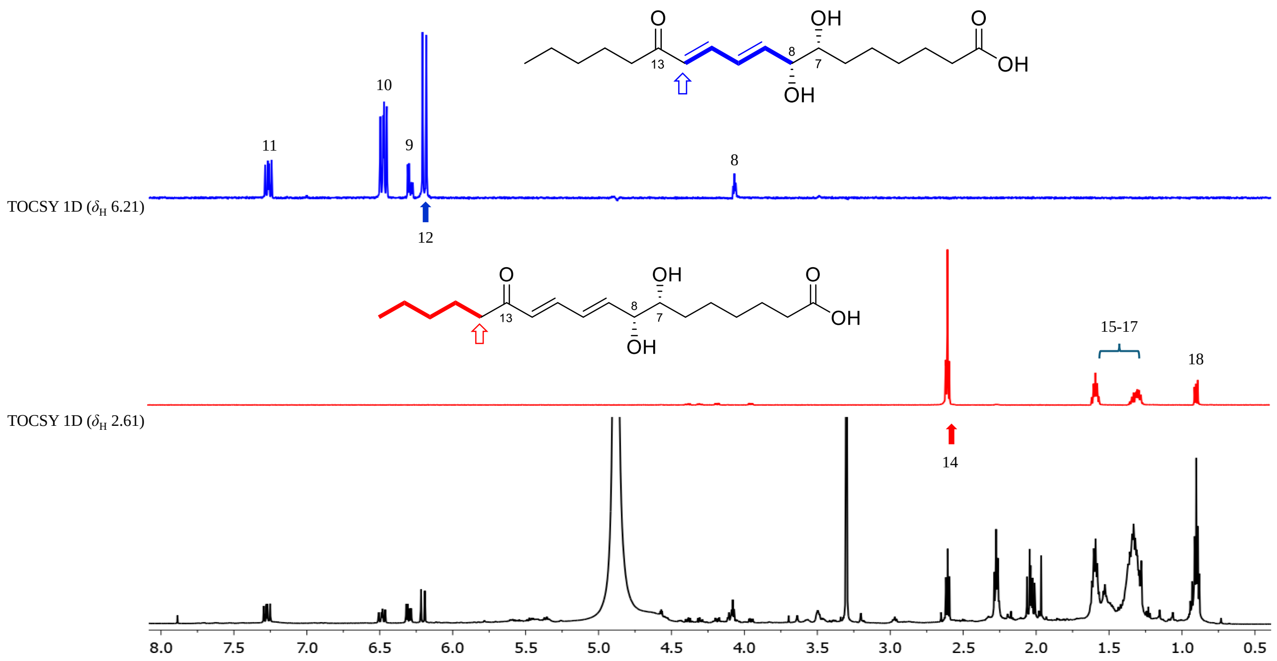

Figure S 18. ^1H-^1H TOCSY spectrum of compound (**2**) in CD_3OD

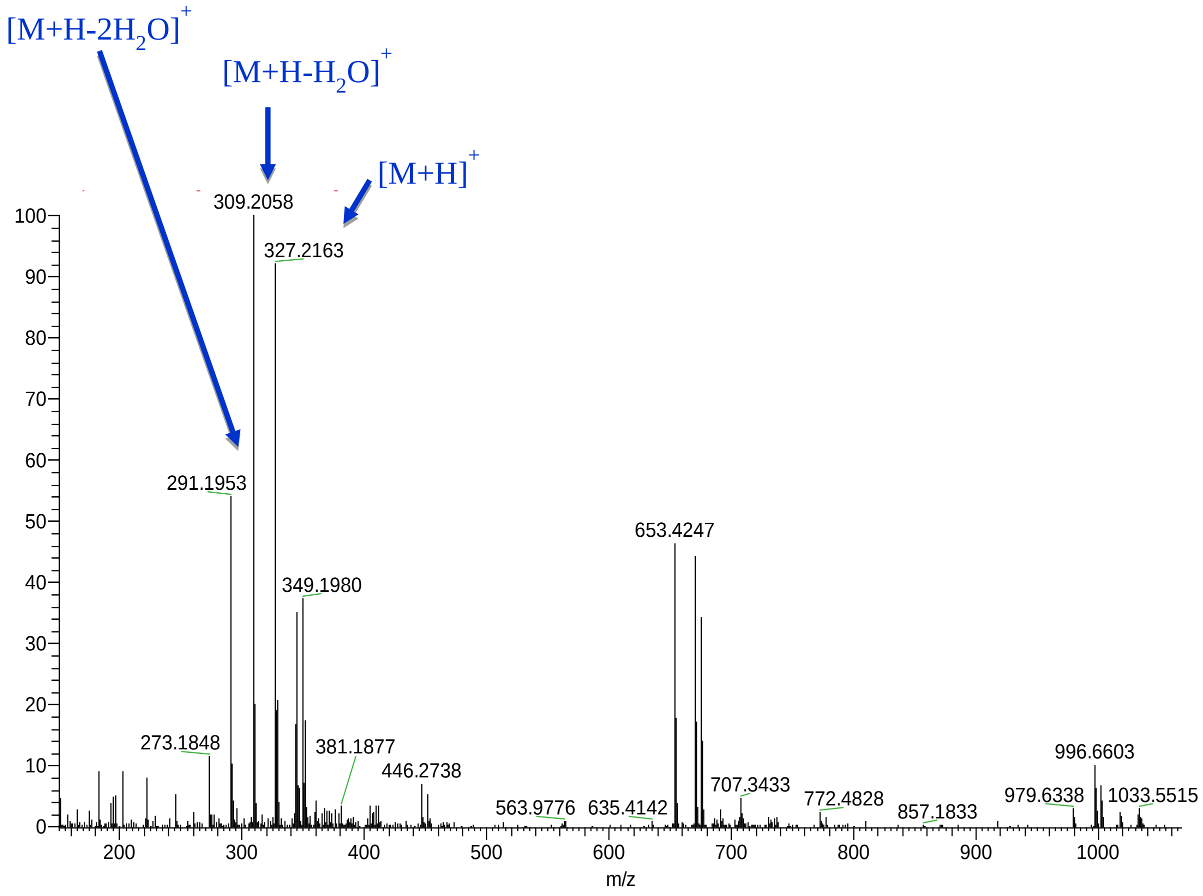

Figure S 19. HRESIMS spectrum of compound (**2**)

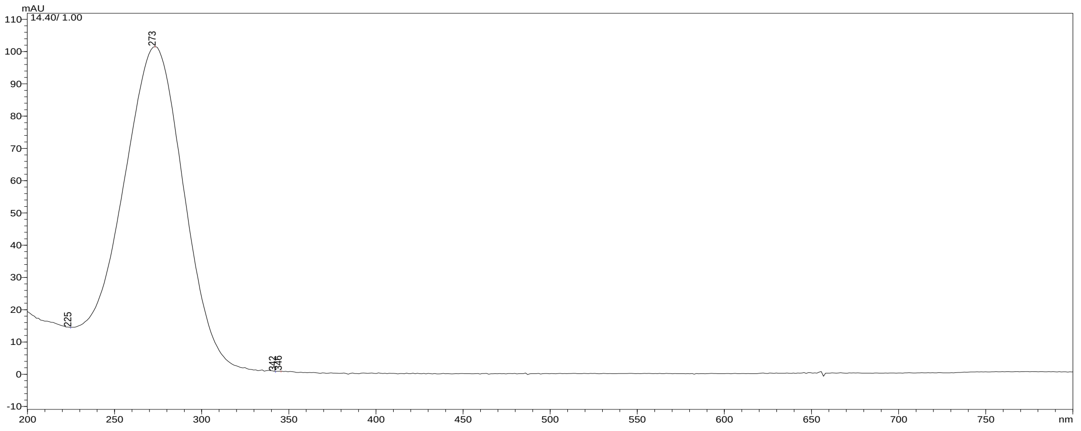

Figure S 20. UV spectrum (1µg/mL, MeOH) of (**2**)

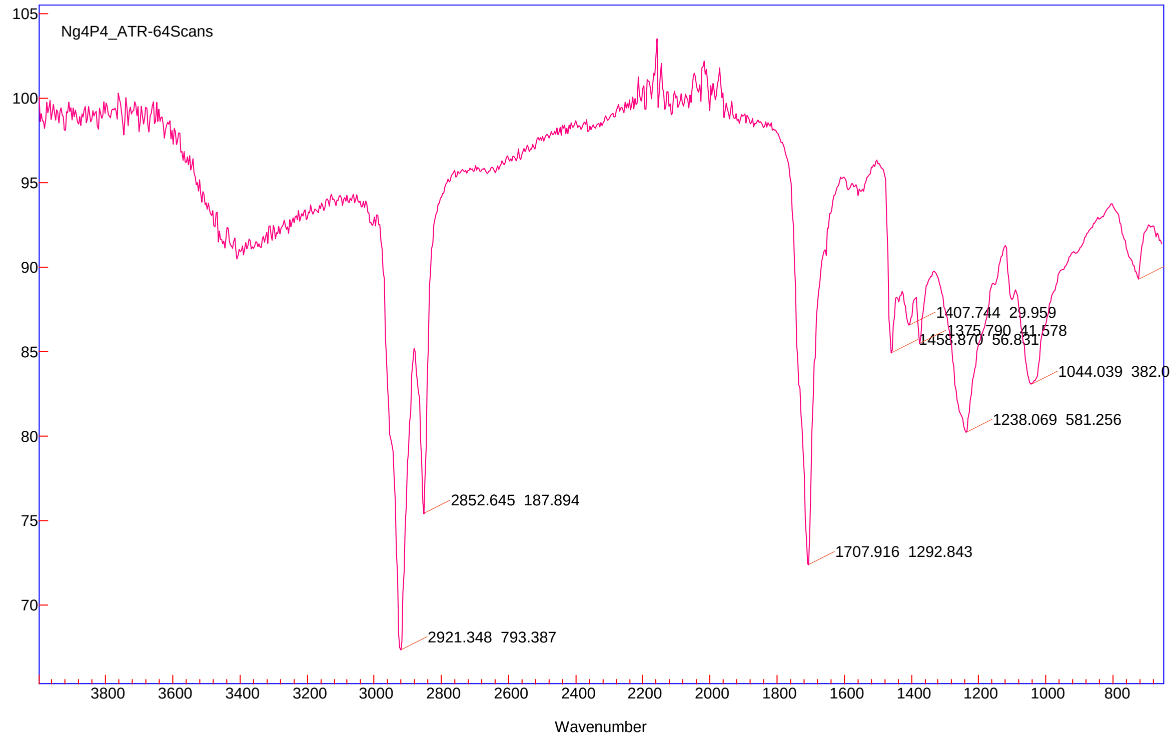

Figure S 21. IR spectrum recorded by of compound (**3**) using liquid solution

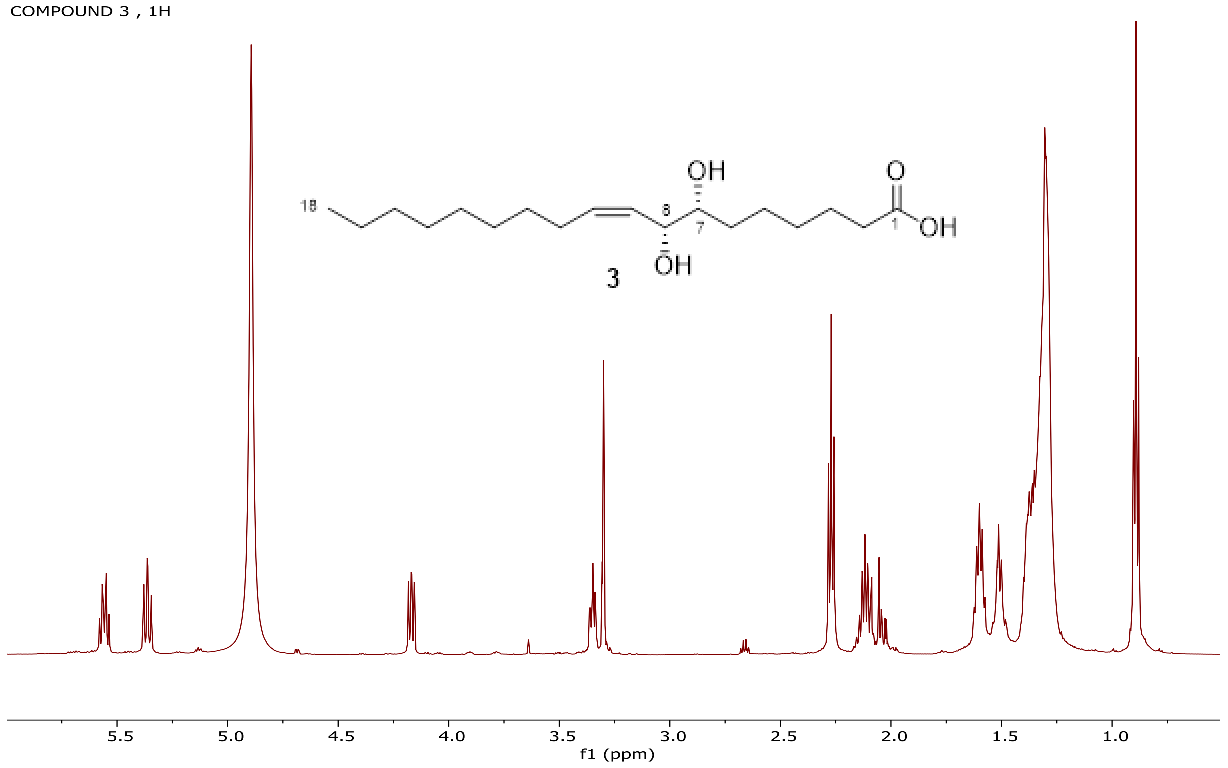

Figure S 22. ^{1}H NMR (600 MHz) spectrum of compound (**3**) in CD_3OD

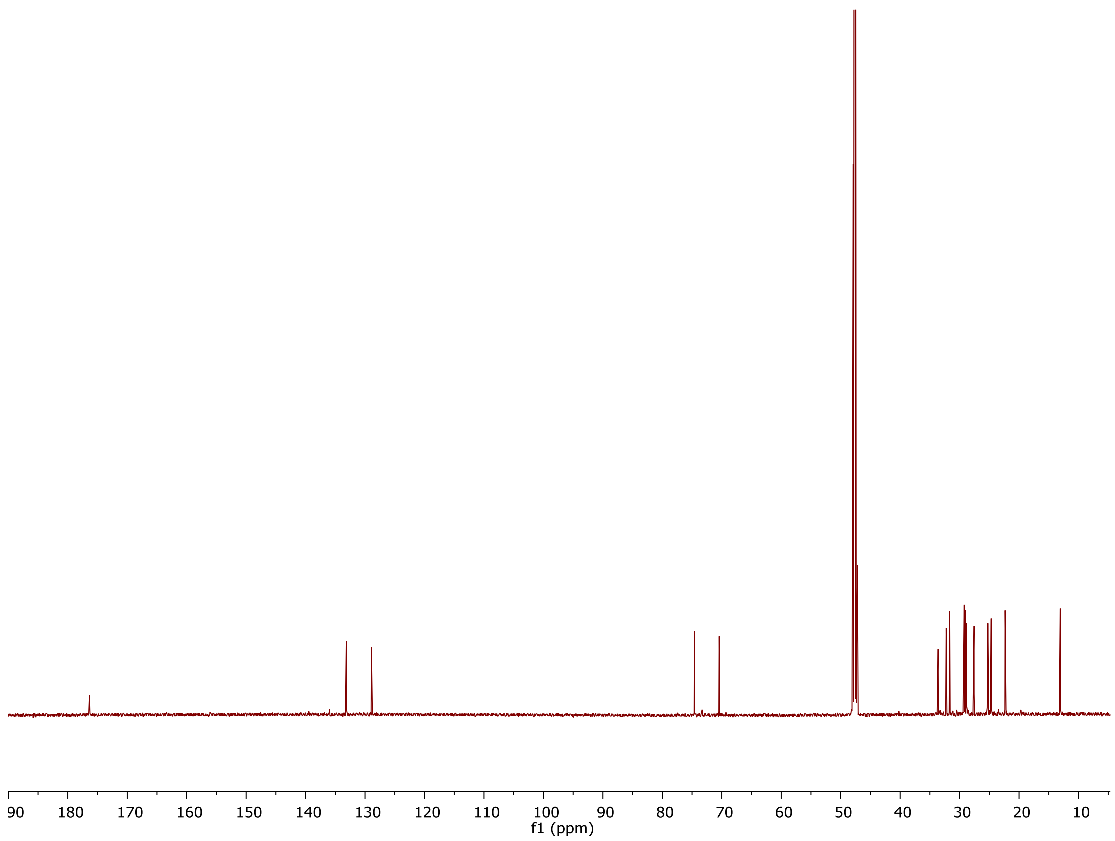

Figure S 23. ^{13}C NMR (150 MHz) spectrum of compound (**3**) in CD_3OD

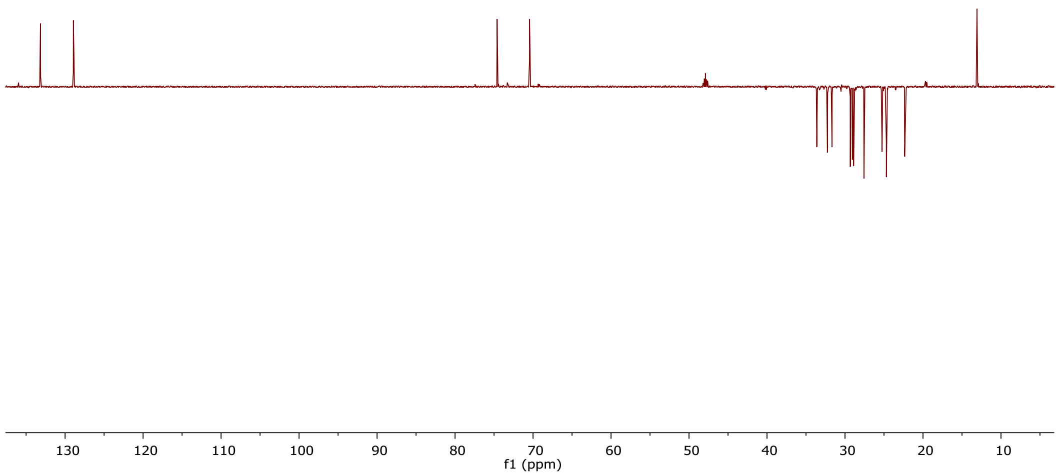

Figure S 24.^{13}C NMR and DEPT 135 (150 MHz) spectrum of compound (**3**) in CD_3OD

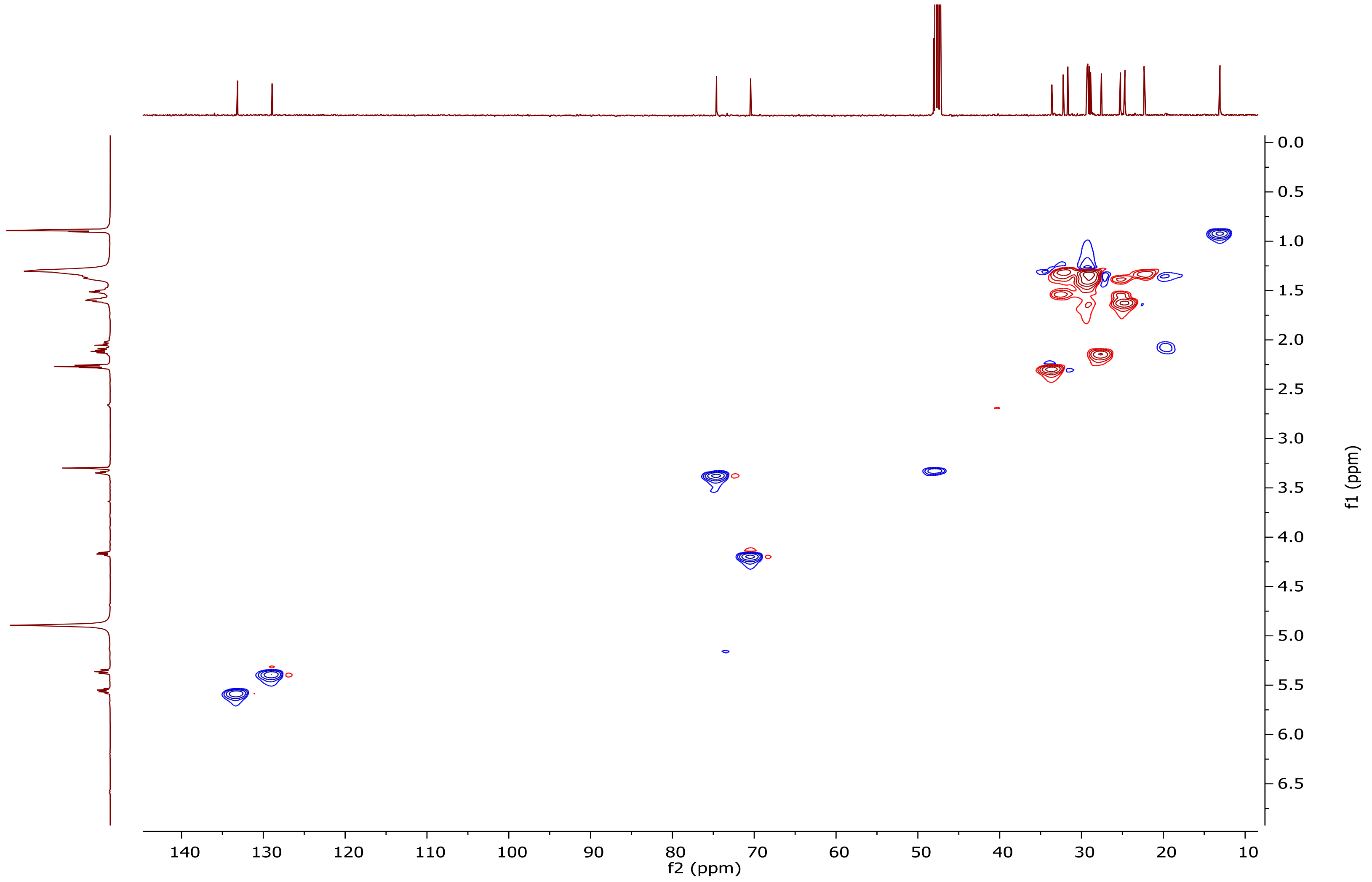

Figure S 25. HSQC spectrum of compound (**3**) in CD_3OD

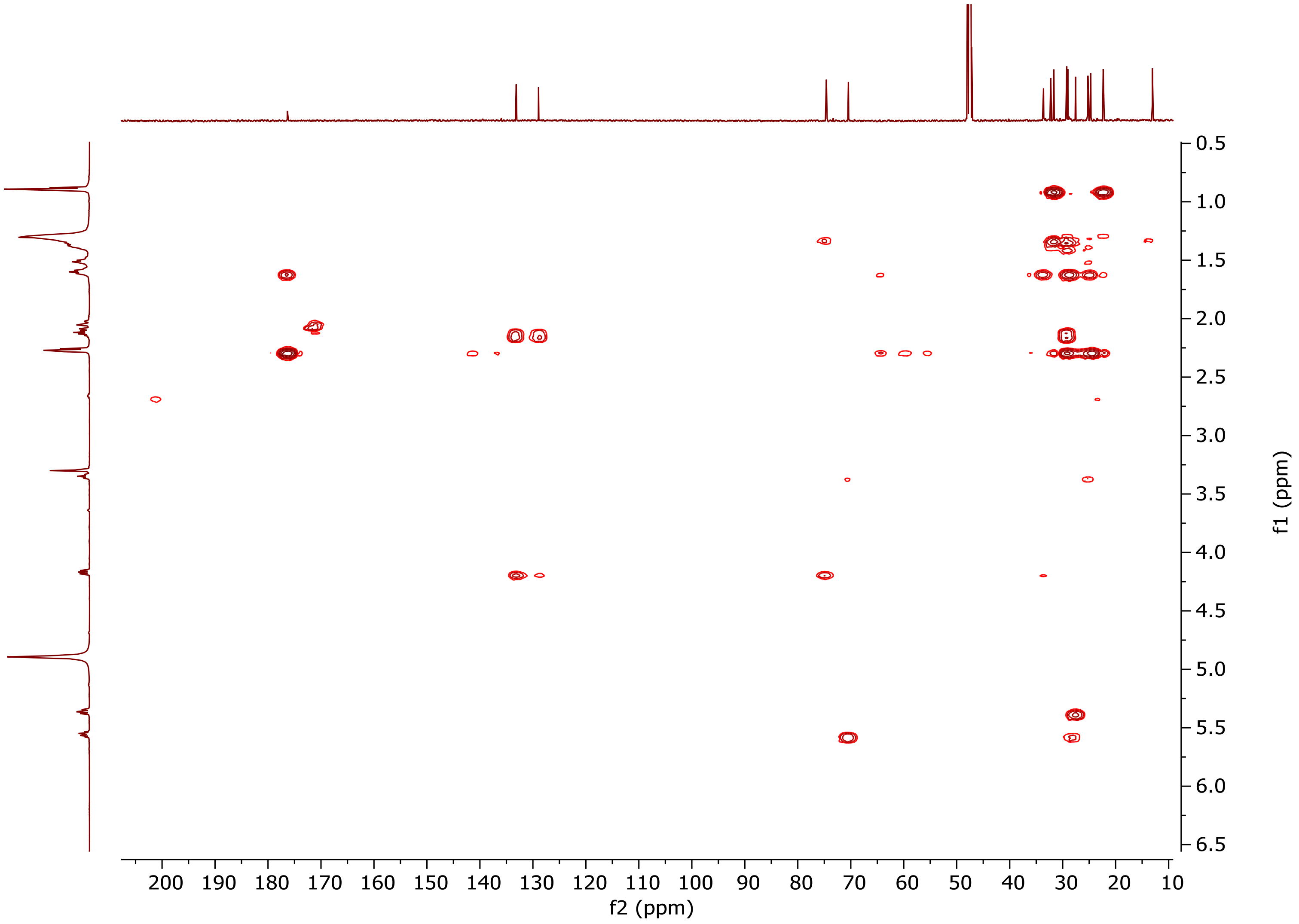

Figure S 26. HMBC spectrum of compound (**3**) in CD_3OD

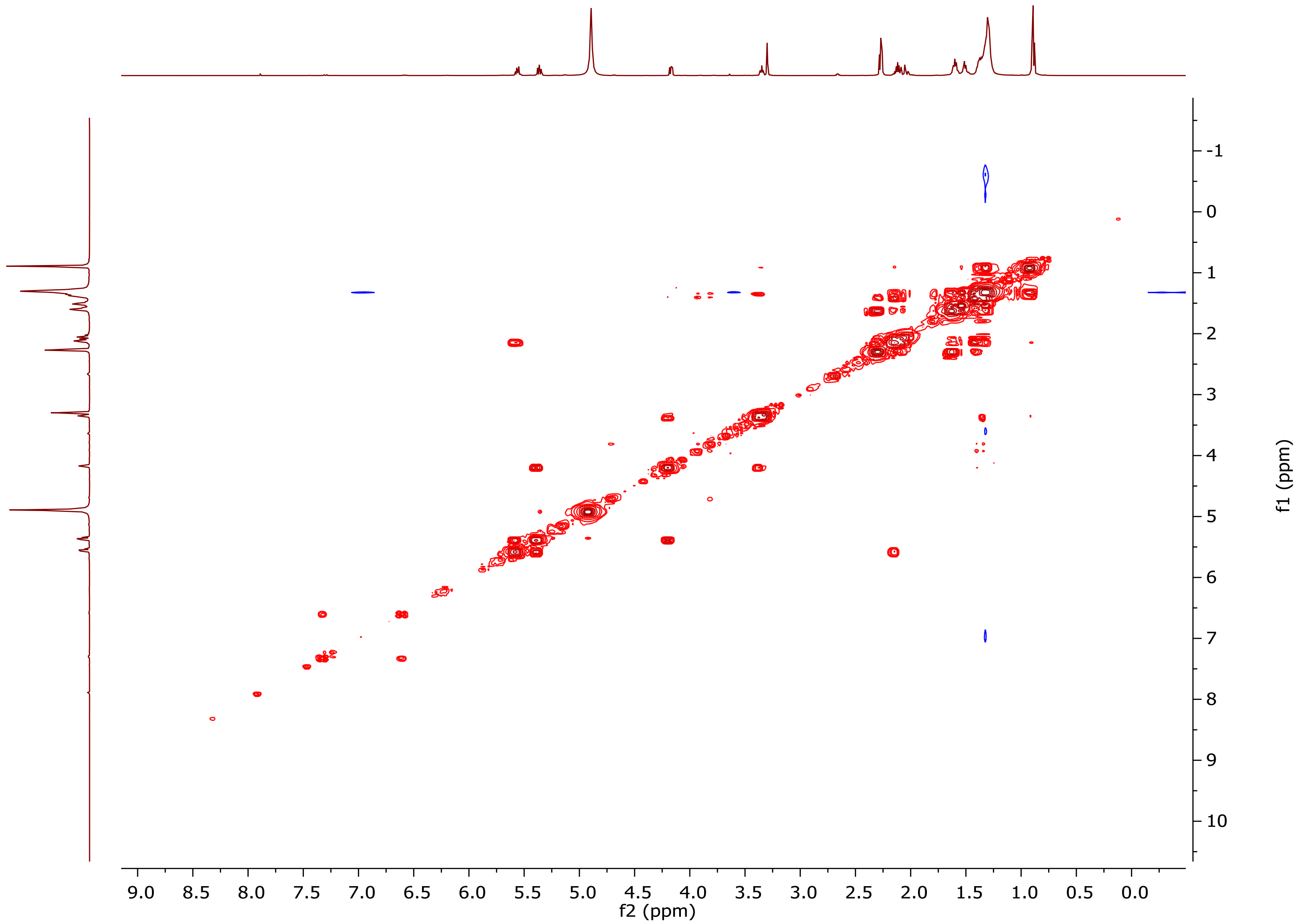

Figure S 27. ^1H-^1H COSY spectrum of compound (**3**) in CD_3OD

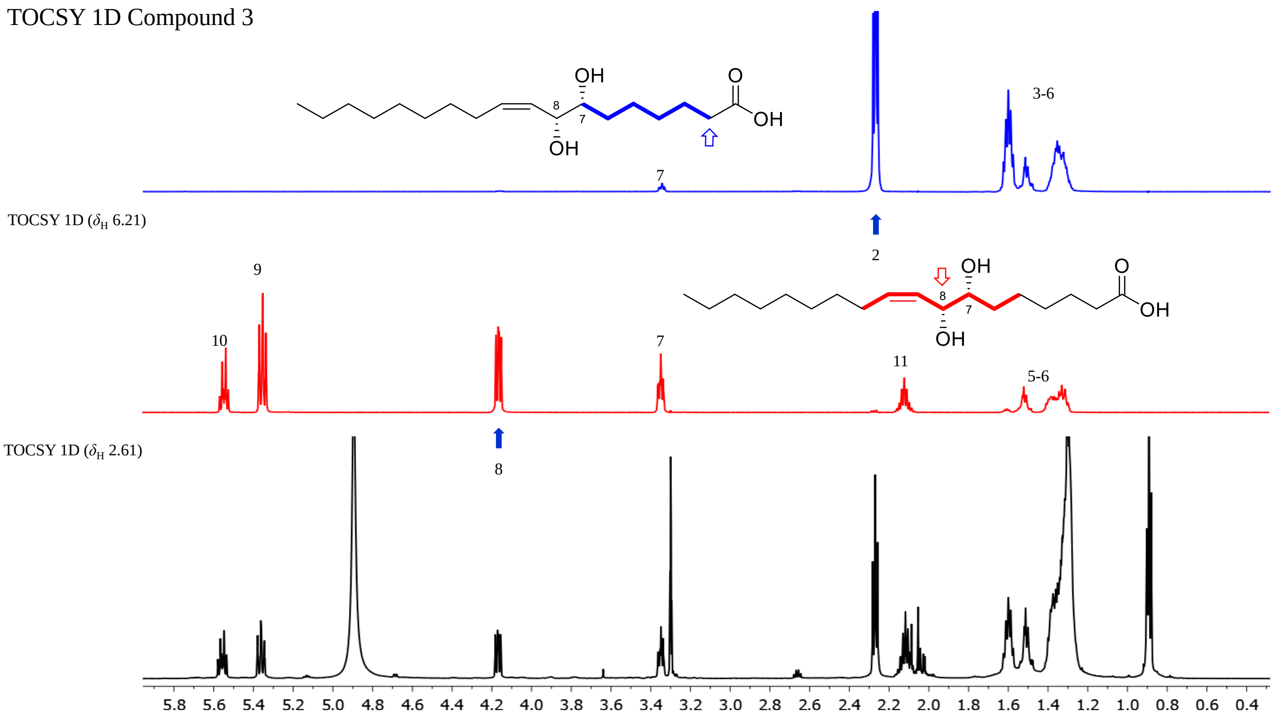

Figure S 28. ^{1}H-^{1}H TOCSY spectrum of compound (**3**) in CD_3OD

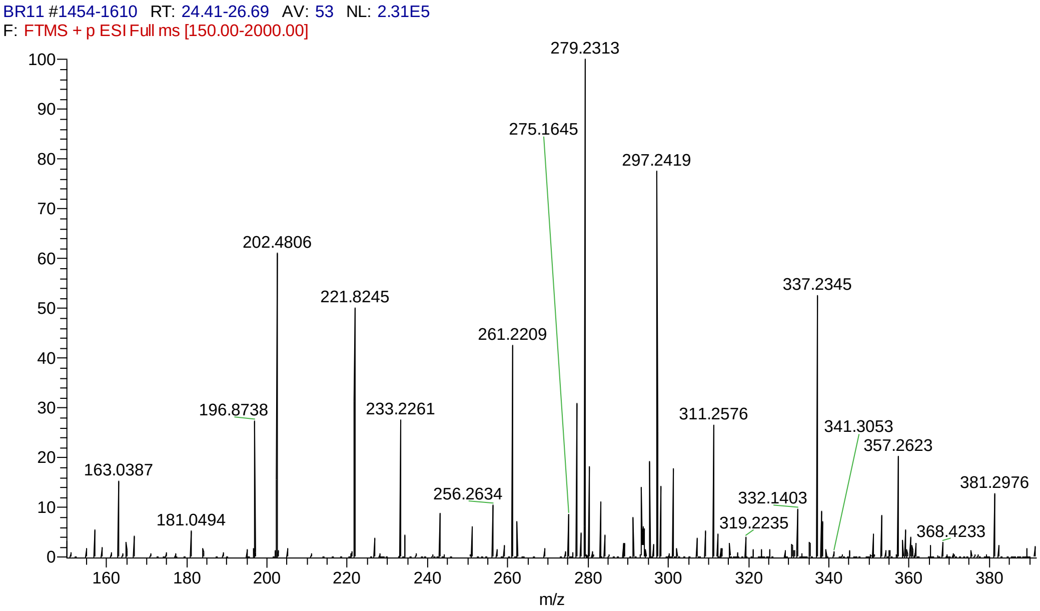

Figure S 29. HRESIMS spectrum of compound (**3**)

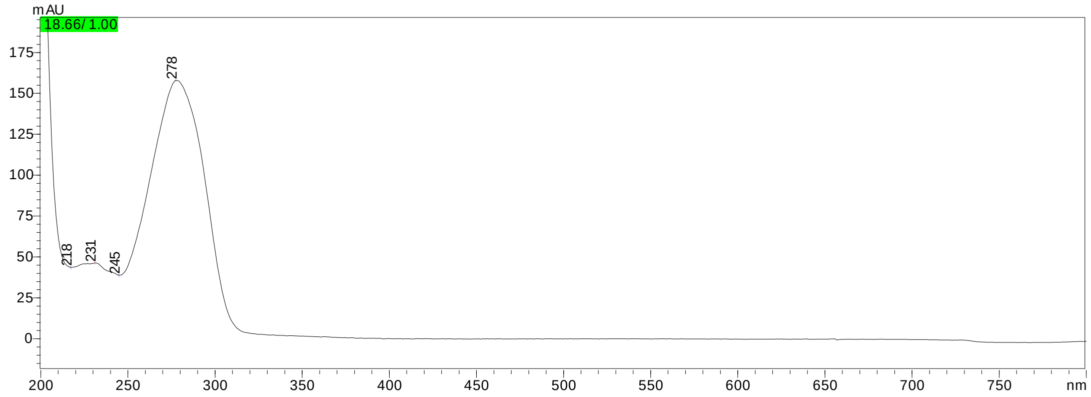

Figure S 30. UV spectrum (1µg/mL, MeOH) of (**3**)

List of Supplementary Tables

COMPOUND 1

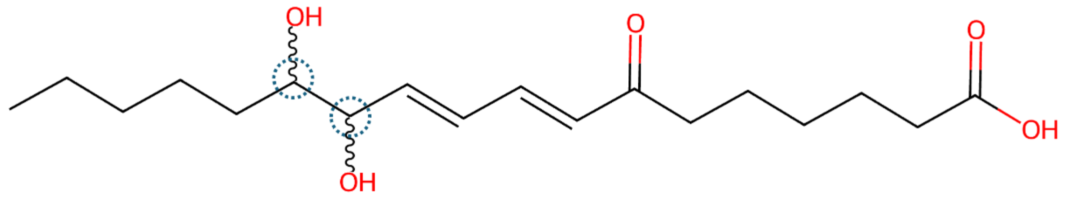

Semi-empirical pre-optimization: PM6 Optimization with DFT: b3lyp/6-311+G*

Table 1: Relative Energies, Enthalpy and Gibbs free energy (kcal/mol).

	RR	SR	SS	RS
**Energy**	-677653.51	-677652.736	-677653.51	-677652.736
**Enthalpy**	-677347.019	-677346.388	-677347.019	-677346.388
**Gibbs free energy**	-677406.717	-677406.114	-677406.717	-677406.114

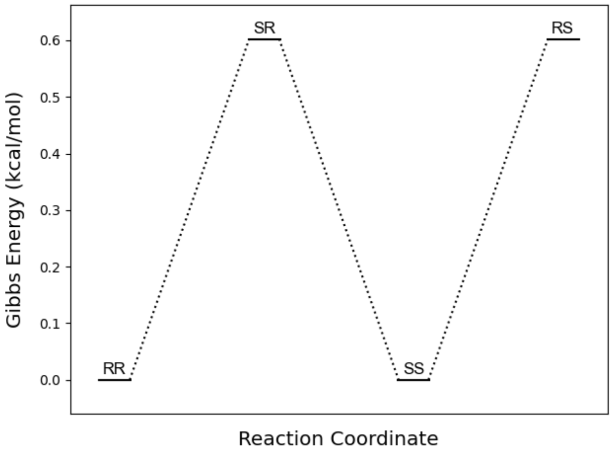

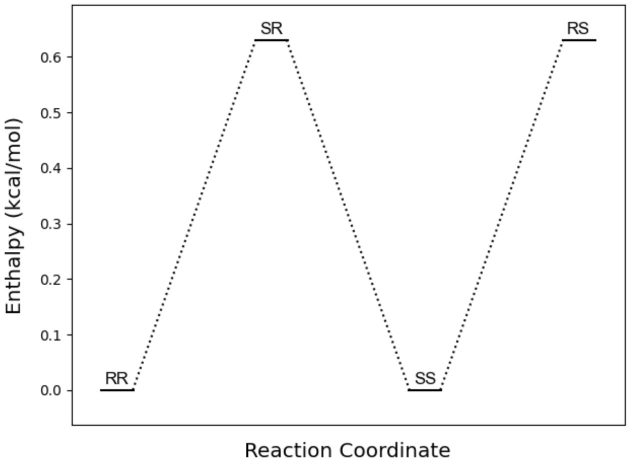

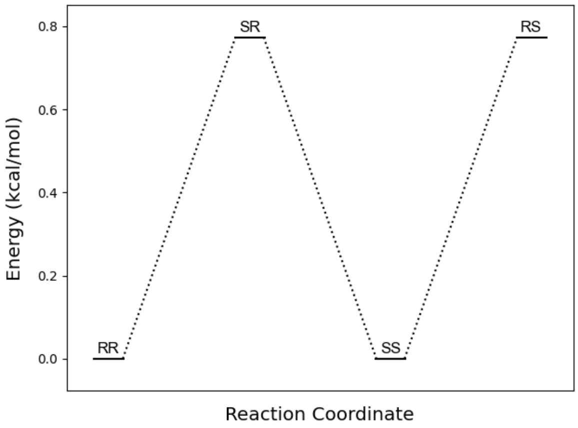

RR	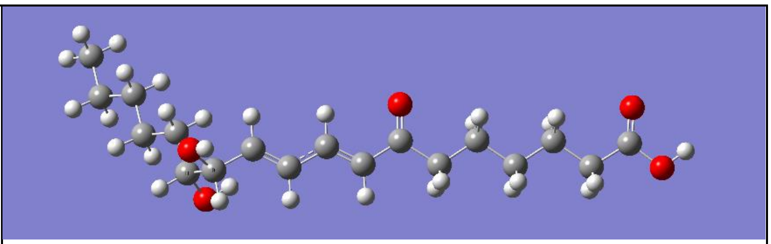
SR	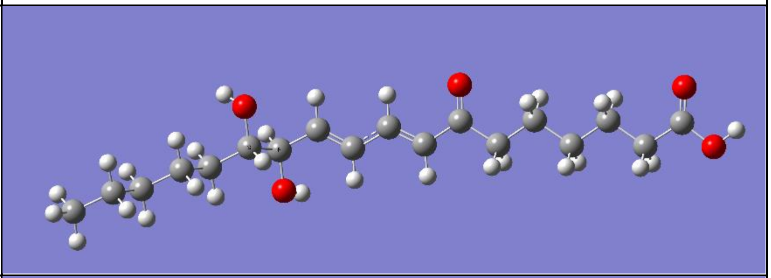
SS	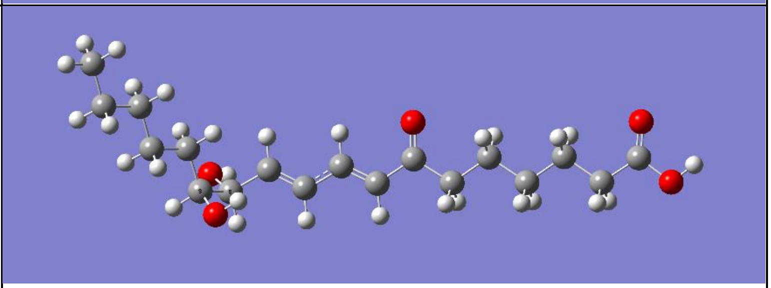
RS	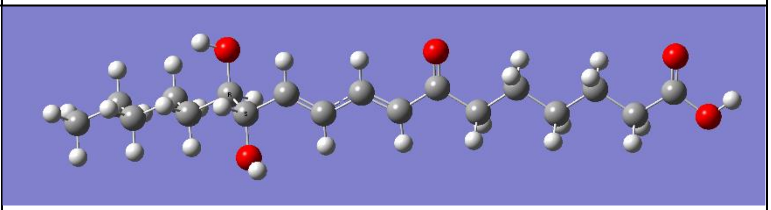

COMPOUND 2

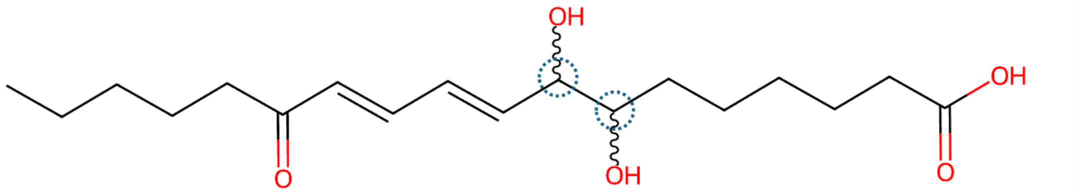

Semi-empirical pre-optimization: PM6 Optimization with DFT: b3lyp/6-311+G*

Table 2: Relative Energies, Enthalpy and Gibbs free energy (kcal/mol).

	**RR**	**SR**	**SS**	**RS**
**Energy**	-677651.807	-677651.898	-677653.013	-677652.043
**Enthalpy**	-677345.164	-677345.26	-677346.285	-677345.448
**Gibbs free energy**	-677404.806	-677404.715	-677406.013	-677405.149

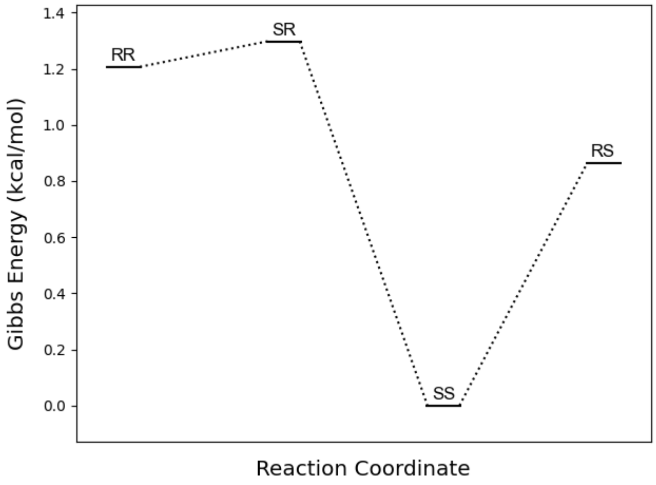

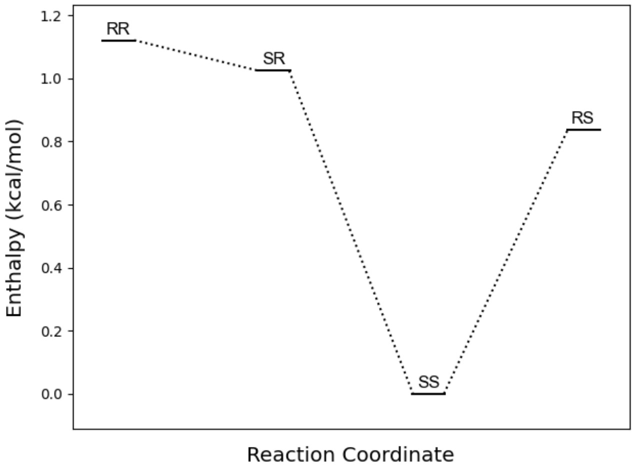

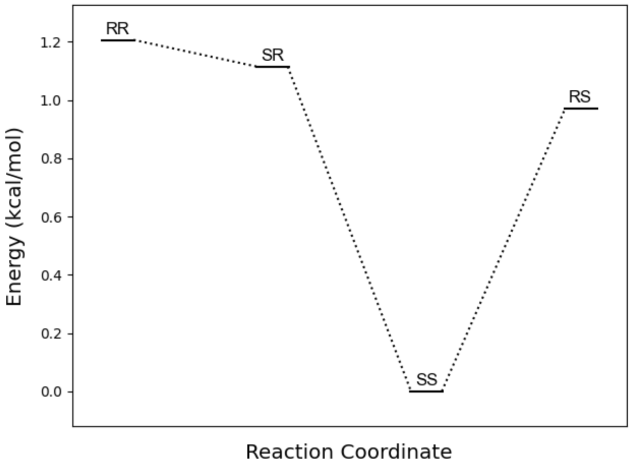

RR	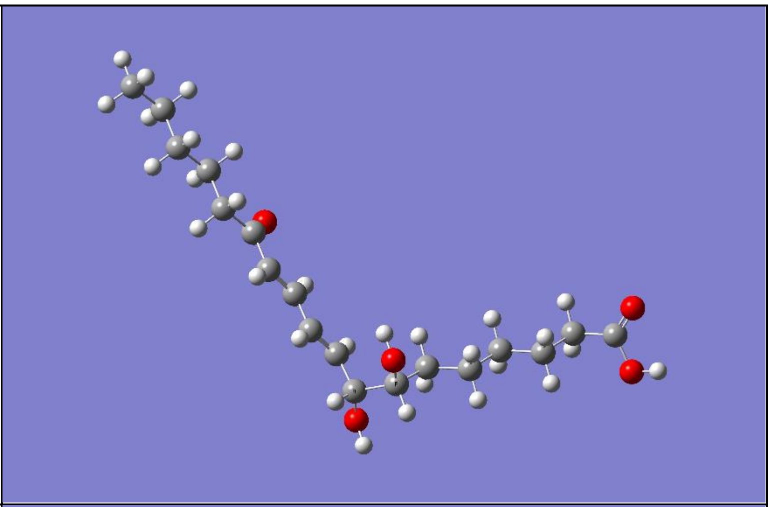
SR	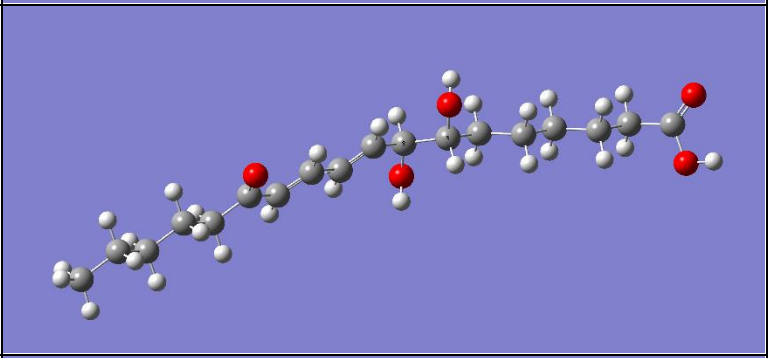
SS	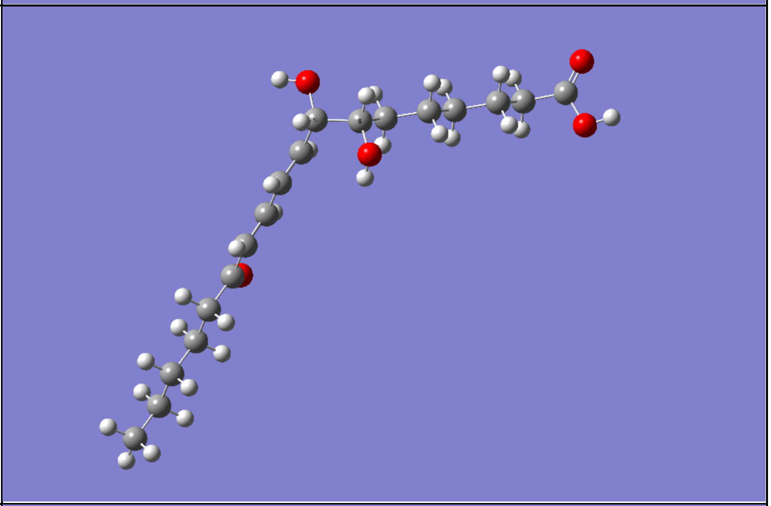
RS	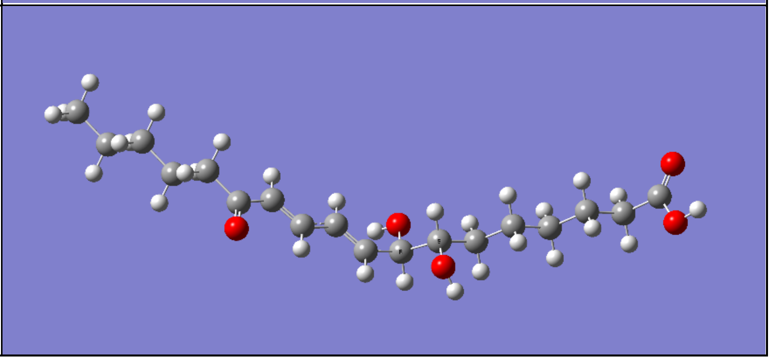

COMPOUND 3

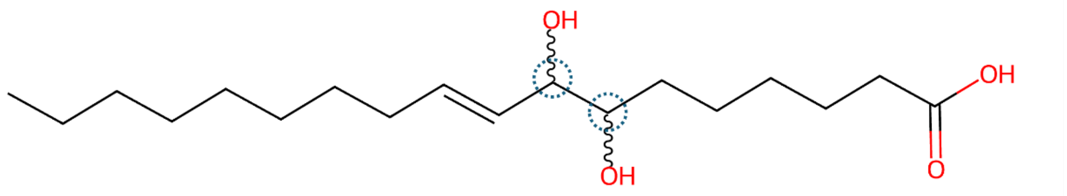

Semi-empirical pre-optimization: PM6 Optimization with DFT: b3lyp/6-311+G*

Table 3: Relative Energies, Enthalpy and Gibbs free energy (kcal/mol).

	RR	SR	SS	RS
**Energy**	-631930.808	-631929.072	-631930.808	-631929.072
**Enthalpy**	-631597.664	-631596.102	-631597.664	-631596.102
**Gibbs free energy**	-631656.741	-631655.352	-631656.741	-631655.352

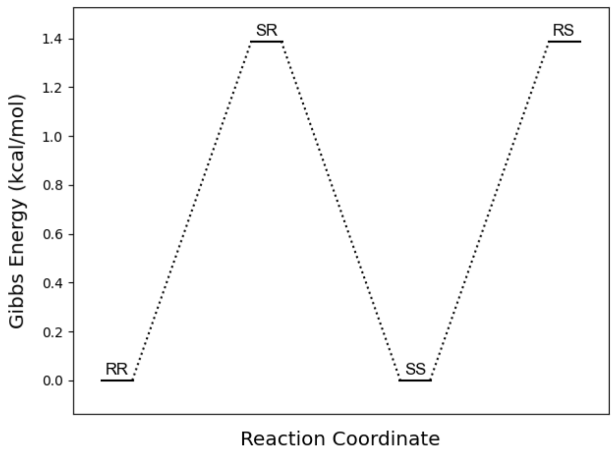

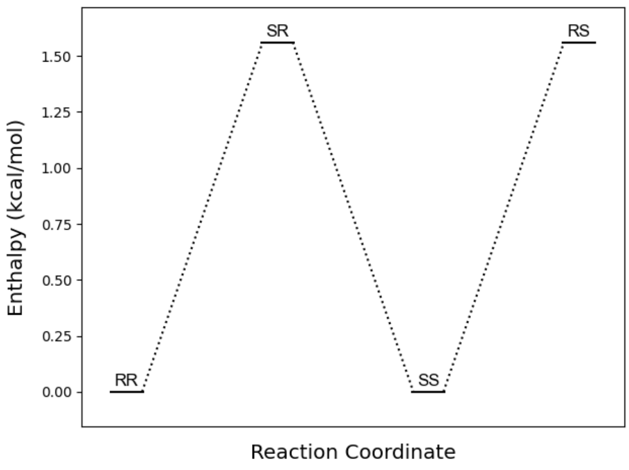

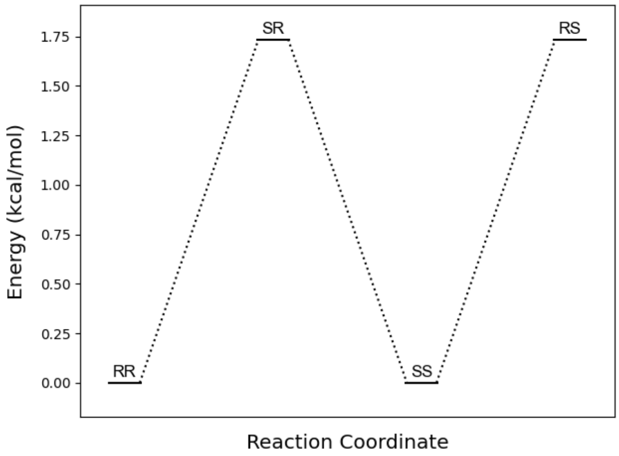

RR	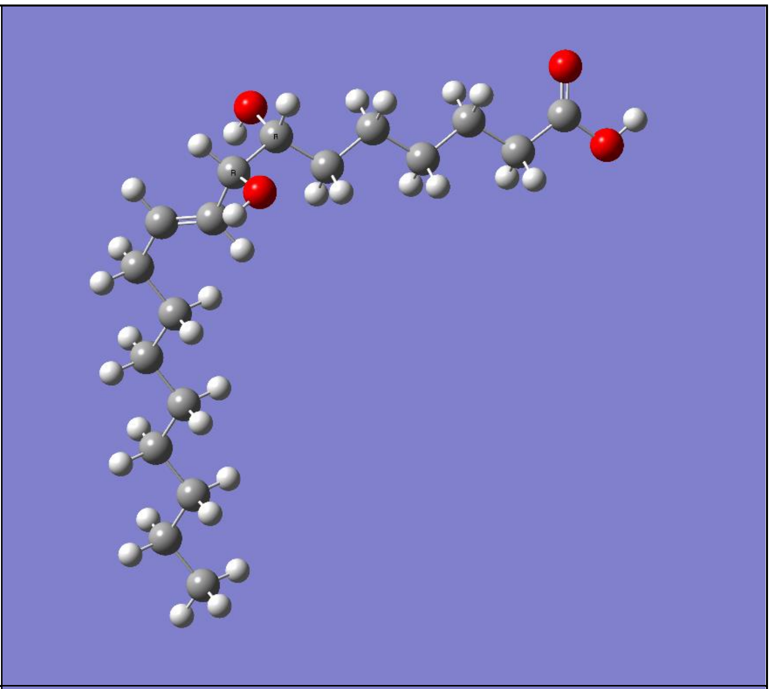
SR	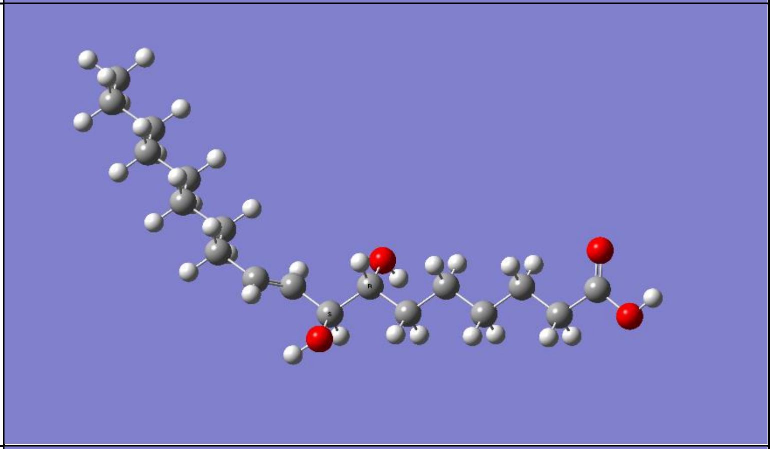
SS	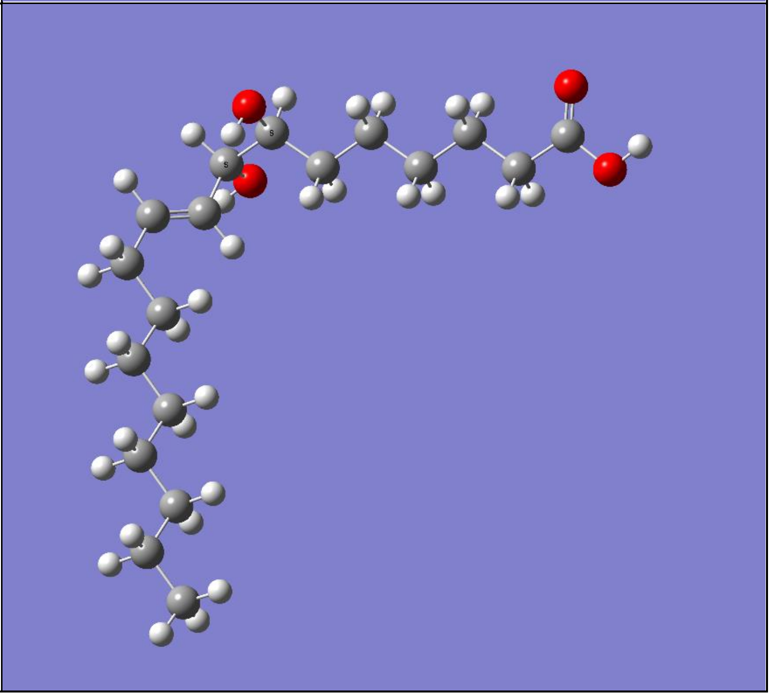

RS	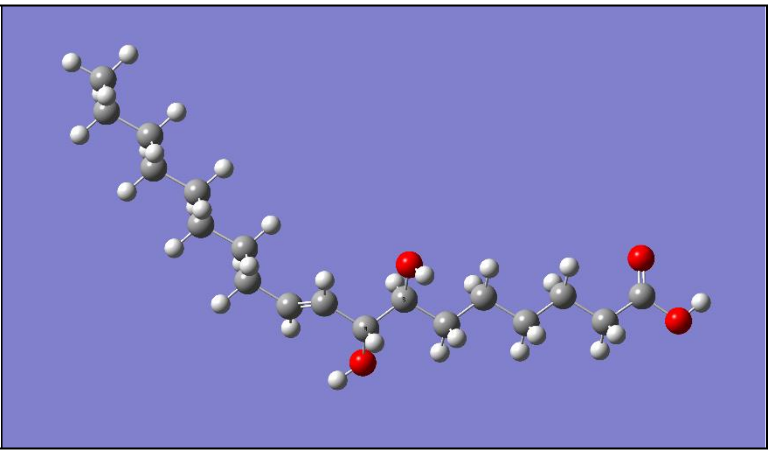
